# A comparison of three mucus-secreting airway cell lines (Calu-3, SPOC1 and UNCN3T) for use as biopharmaceutical models of the nose and lung

**DOI:** 10.1016/j.ejpb.2021.07.016

**Published:** 2021-10

**Authors:** Diane F. Lee, Michael I. Lethem, Alison B. Lansley

**Affiliations:** a School of Pharmacy and Biomolecular Sciences, University of Brighton, Brighton BN2 4GJ, UK; b School of Veterinary Medicine, University of Surrey, Guildford GU2 7AL, UK^1^

**Keywords:** Mucus barrier, Mucin, Permeability barrier, Cell-based assay, Drug absorption, Nasal drug delivery, Airway epithelial cells, Pulmonary drug delivery, Irritancy, Testosterone, AMP-PNP, Adenylyl-imidodiphosphate, ATP, Adenosine triphosphate, Papp, Apparent Permeability Coefficient, DMEM, Dulbecco’s Modified Eagle’s Mediun, TEER, Transepithelial Electrical Resistance, TJ, Tight junction, AJ, Adherens junction, ZO-1, Zonula occludens-1, MD, Mucus-depleted, ALI, Air–liquid interface, qRT-PCR, Quantitative real-time polymerase chain reaction, ELLA, Enzyme-linked lectin assay, ELISA, Enzyme-linked immunosorbent assay, FD4, Fluorescein isothiocyanate dextran average molecular weight 4000 Da, IL-6, Interleukin 6, IL-8, Interleukin 8, TNF-α, Tumour necrosis factor-α, IFN-γ, Interferon-γ, TB, Transport buffer

## Abstract

The aim of this work was to compare three existing mucus-secreting airway cell lines for use as models of the airways to study drug transport in the presence of mucus.

Each cell line secreted mature, glycosylated mucins, evidenced by the enzyme-linked lectin assay. The secretagogue, adenylyl-imidodiphosphate, increased mucin secretion in SPOC1 (3.5-fold) and UNCN3T (1.5-fold) cells but not in Calu-3 cells. In a novel mucus-depleted (MD) model the amount of mucus in the non-depleted wells was 3-, 8- and 4-fold higher than in the mucus-depleted wells of the Calu-3, SPOC1 and UNCN3T cells respectively. The permeability of 'high mucus’ cells to testosterone was significantly less in SPOC1 and UNCN3T cells (P < 0.05) but not Calu-3 cells.

Mucin secretion and cytokine release were investigated as indicators of drug irritancy in the SPOC1 and UNCN3T cell lines. A number of inhaled drugs significantly increased mucin secretion at high concentrations and the release of IL-6 and IL-8 from SPOC1 or UNCN3T cells (P < 0.05).

SPOC1 and UNCN3T cell lines are better able to model the effect of mucus on drug absorption than the Calu-3 cell line and are proposed for use in assessing drug-mucus interactions in inhaled drug and formulation development.

## Graphical abstract

**Figure:**
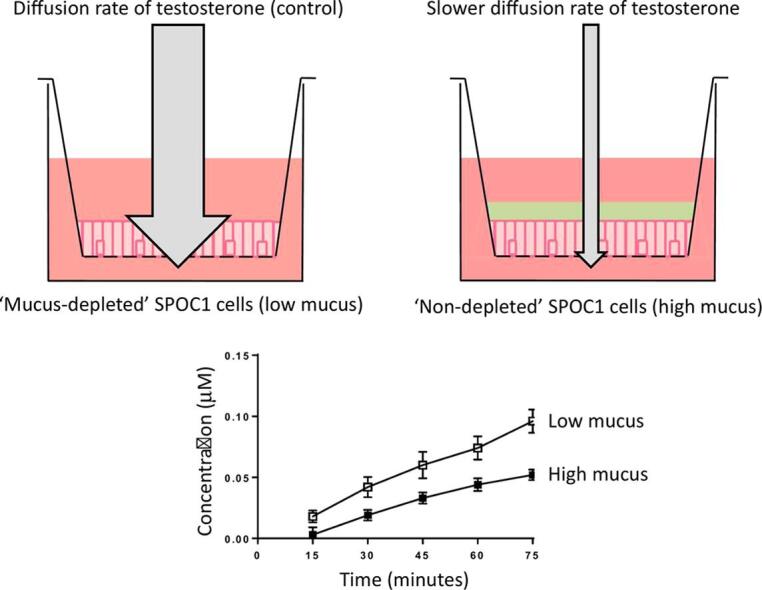

## 1 Introduction

Mucus is a vital component of homeostasis in the upper airways, which extend from the nose to the terminal bronchioles, forming the first line of defence against pathogens and irritants in its function as a chemical, physical and immunological barrier [1]. However, mucus may also act as a barrier to drug and particulate absorption [2], [3], particularly in cases of mucus hypersecretion, a characteristic of chronic inflammatory airway diseases such as chronic bronchitis, bronchiectasis, severe asthma and cystic fibrosis (CF). Therefore, it is logical that *in vitro* cell culture models of the airways should secrete mucus.

The effect of mucus on drug dissolution and absorption is poorly understood due to the lack of models that accurately reflect the native epithelium [4], [5], [6]. The potential for improved pharmacokinetic profiles and continued interest in targeting the nose and lung for delivery of drugs to the (i) central nervous system (nose), (ii) systemic circulation and (iii) the local treatment of respiratory disorders is driving the need for new *in vitro* models to study drug absorption across the airway epithelium. It is pertinent to consider the presence of mucus in such studies [7], [8], [9]. The permeability of several human cell lines e.g. 16HBE14o- and Calu-3, which demonstrate a respiratory bronchial epithelial cell-like phenotype and the formation of adequate tight junctions, to drugs and particulates has been studied previously [7], [10], [11]. Such models also simulate the nasal epithelium since the bronchial and nasal epithelium are similar [12]. However, such studies rarely consider the effect of mucus as an additional permeability barrier.

A variety of different models has been used to consider the role of mucus in the dissolution or absorption of drugs or drug delivery systems and are the subject of reviews by Lock et al. [6] and Lechanteur et al. [9]. One approach is to supplement non-mucus secreting cell lines with a layer of mucus [13]. However, it is challenging to achieve an even layer of mucus of the appropriate dimensions. Perhaps the best *in vitro* models currently available are those using cells capable of secreting mucus either primary [14], [15] or cell lines such as Calu-3 [16], [17], [18].

The principal aim of the current study was to explore the potential of the mucus-secreting cell lines SPOC1 and UNCN3T as drug absorption models of the airways and to compare them to the Calu-3 cell line, which is widely used to model the airways.

SPOC1 cells, spontaneously immortalised from rat tracheal epithelial cells, have previously been shown to secrete mucins, detected by Alcian blue and periodic acid-Schiff staining and the enzyme-linked lectin assay (ELLA), in response to purinergic agonists [19], [20], [21], [22]. When cultured at an air–liquid interface (ALI), SPOC1 cells develop a transepithelial electrical resistance (TEER) indicative of a polarised epithelium [19].

The UNCN3T cell line is one of six cell lines first described in 2009 [23], which were created to overcome the need for primary airway cells and to counter some of the disadvantages of primary cultures, such as their high variability and expense. The creators compared each of the six cell lines with their parent cells and UNCN3T was described as useful for studies requiring mucous secretory cell differentiation and function [23]. Together with their human origin, demonstrable normal morphology and measurable TEER of > 200 Ω.cm^2^, these qualities make UNCN3T cells a promising prospect as a model to study drug absorption.

In contrast to Calu-3 cells, there are no published studies of the use of SPOC1 and UNCN3T cells in drug absorption studies. We therefore further characterised these two cell lines and compared them to the Calu-3 cell line. Mucin secretion from all three cell lines was characterised using quantitative real-time polymerase chain reaction (qRT-PCR) in conjunction with the ELLA. It is well established that extracellular ATP is a potent stimulus for regulated mucus secretion via purinergic receptors [24], [25], [26]. Moreover, it is possible to enhance mucin secretion in certain models of the airway epithelium using ATP or its non-hydrolysable analogue, AMP-PNP [27], [28], [29]. Therefore, the effect of mucus on drug absorption can be studied by comparing drug absorption in the presence of normal mucus levels and mucus levels increased by ATP (or AMP-PNP). This was the first approach tested in all three cell lines (Secretagogue model). An alternative approach was also taken where drug absorption in the presence of accumulated mucus levels was compared to drug absorption in the presence of depleted mucus levels (as a consequence of washing the cultures) (mucus-depleted (MD) model). With both approaches, the aim was to maximise the difference in mucin levels between ‘low mucus’ and ‘high mucus’ cultures and, as a result, the sensitivity of the model. The models were validated by assessing the ability of the secreted mucus to act as a barrier to the absorption of testosterone. Testosterone was selected as a model compound, rather than using inhaled drugs, as the presence of mucus is known to reduce the diffusion of this compound [30].

A secondary aim of the study was to see if the models could predict irritancy in the airways. It is important to be able to predict the local side effects of inhaled drugs, to avoid the exacerbation of disease and to streamline the drug discovery process. Increased mucus secretion may be expected to predict irritancy, as it occurs in response to inhaled irritants such as cigarette smoke [31], [32], or allergens such as grass pollen [1]. Further, the Slug Mucosal Irritation assay has used increased mucus production to predict nasal stinging, itching and burning sensations [33], [34]. The second proposed measure of irritancy in the airways is cytokine release. Cytokines are routinely used as measures of irritancy and are also linked to mucus production in the airways [35], [36].

In summary, the principal aim of this study was to establish whether the mucus-secreting *in vitro* models of the airways (Calu-3, SPOC1 and UNCN3T) were sufficiently sensitive to reveal an effect of mucus on the transport of the model compound, testosterone. A secondary aim was to determine whether the *in vitro* models could be used to study drug irritancy of the airways, using cytokine release and/or mucin secretion as the output data.

Demonstration of these criteria would indicate the suitability of the model for use in drug development studies of novel inhaled therapeutics.

## 2 Materials and methods

### 2.1 Materials

Testosterone, testosterone-d_3_ and fluorescein isothiocyanate dextran, average molecular weight 4000 Da (FD4) were purchased from Sigma Aldrich (Sigma Aldrich, Gillingham, UK) with ≥ 98% purity. Also purchased from Sigma Aldrich were HPLC-grade methanol. Ipratropium bromide monohydrate, tiotropium bromide monohydrate, budesonide and salbutamol sulfate were kindly supplied by GlaxoSmithKline, Stevenage, UK). Deionised water was used throughout.

### 2.2 Calu-3 cell culture

Prior to seeding Calu-3 cells (LGC Standards, Teddington, UK) onto permeable culture supports, the cells were maintained in Dulbecco’s Modified Eagles Medium (DMEM), high glucose with pyruvate, supplemented with 10 % foetal bovine serum (FBS), 1 % non-essential amino acids and 1 U mL^−1^ penicillin/1 μg mL^−1^ streptomycin (all PAA Laboratories, GE Healthcare, Buckinghamshire, UK) [29] (Calu-3 maintenance medium). Cells were cultured in a humidified 5 % CO_2_/95 % air environment at 37 °C. Medium was changed every 2–3 days and passage performed at ~ 80 % confluence. Calu-3 cells (passage numbers 19-45) were seeded onto uncoated Transwell-Clear™ inserts (12 mm, 0.4 μm pore size) (Corning Costar, Cambridge, MA) at a density of 5 × 10^5^ cells/well. Following three days of submerged culture, the cells were cultured at air–liquid interface (ALI) for 18-21 days before use in mucin secretion assays and permeability experiments.

### 2.3 SPOC1 cell culture

SPOC1 cells were a kind gift from the Cystic Fibrosis/Pulmonary Research and Treatment Centre, University of North Carolina, NC, US. Cells were cultured according to the methods of Abdullah *et al.* [20]. Prior to seeding onto permeable culture supports, cells were maintained in 1:1 DMEM:Ham’s F12 medium with glutamine (Gibco Life Technologies, Thermo Fisher Scientific, Glasgow, UK), supplemented with 2 % v/v FBS, 5 ng mL^−1^ epithelial growth factor (BD Biosciences, Oxford, UK), 5 μg mL^−1^ transferrin (Sigma Aldrich), 5 μg mL^−1^ insulin (Sigma Aldrich), 0.1 μg mL^−1^ hydrocortisone (Sigma Aldrich), 0.1 μg mL^−1^ cholera toxin (Sigma Aldrich), 50 μM ethanolamine (Sigma Aldrich), 50 μM phosphoethanolamine (Sigma Aldrich), 1.5 mg mL^−1^ bovine serum albumin (globulin free) (Sigma Aldrich), 70 μg mL^−1^ bovine pituitary extract (BPE) (Sigma Aldrich), 5 × 10^-4^ M retinoic acid (Sigma Aldrich) and 15 mM 4-(2-hydroxyethyl)-1-piperazineethanesulfonic acid (HEPES) (Gibco), pH 7.3 (SPOC1 maintenance medium). Cells were cultured in a humidified 5 % CO_2_/95 % air environment at 37 °C. Medium was changed every 2–3 days and passage performed at ~ 80 % confluence. In initial studies, SPOC1 cells were seeded on Transwell-COL™ inserts [20] at 8 × 10^5^ cells/well, in SPOC1 maintenance medium. However, as medium was observed to accumulate in the apical chamber meaning that the cells were no longer at an ALI, culture conditions were changed and in later experiments the cells were seeded onto Transwell-Clear™ inserts (12 mm, 0.4 μm pore size) coated with 500 μL of a 1/20 dilution of Geltrex™ extracellular matrix substitute (Thermo Fisher Scientific) in DMEM:Ham’s F12 medium. Inserts were left at room temperature for a minimum of 30 min before removing excess Geltrex™ solution and then used immediately for cell seeding. Cells (passages 9–15) were seeded at 8 × 10^5^ cells/well, in SPOC1 maintenance medium. Cells were fed apically and basolaterally (0.5 mL apical, 1.5 mL basolateral) with maintenance medium until confluent (day 5-7) before switching to Pneumacult™ ALI medium (Stemcell Technologies (Grenoble, France) and feeding basolaterally only (ALI) every 2–3 days. Cells seeded on Transwell-COL™ inserts were used for studies of TEER and response to AMP-PNP. Cells seeded on Geltrex™-coated Transwell Clear™ inserts were used to study TEER and for transport experiments at 21 days after raising cultures to ALI.

### 2.4 UNCN3T cell culture

The UNCN3T cell line was a kind gift received from the Cystic Fibrosis/Pulmonary Research and Treatment Centre, University of North Carolina at Chapel Hill School of Medicine, NC, USA.

UNCN3T cells were cultured according to the method of Fulcher *et al.* [23]. For expansion, cells were routinely cultured on rat tail collagen I-coated plates (Invitrogen, Thermo Fisher Scientific) in bronchial epithelial growth medium (BEGM), adding SingleQuot™ kit components (Lonza Biologics, Slough, UK), with the exception of gentamicin/amphotericin B, to bronchial epithelial basal medium (BEGM+). Cells were cultured in a humidified 5 % CO_2_/95 % air environment at 37 °C. Medium was changed every 2–3 days and passage performed at ~ 80 % confluence.

Transwell-Clear™ inserts (12 mm, 0.4 μm pore size) coated with collagen IV (SigmaAldrich) or Transwell-Clear™ inserts coated with Geltrex™ (as described above) were seeded with cells (passages 9-14), at 5 × 10^5^ cells/well in BEGM+. At confluence (5-7 days), cells were raised to ALI as described previously, using Pneumacult™ ALI medium. Experiments were performed 21 days after switching to ALI.

UNCN3T cells were cultured on collagen IV-coated Transwell-Clear™ inserts, according to the method of Fulcher et al. [23] for studies using AMP-PNP. However, UNCN3T cells were also cultured on Geltrex™-coated inserts to enable a direct comparison with the SPOC1 counterpart MD model, in terms of mucin secretion and permeability studies.

### 2.5 Transepithelial electrical resistance of cells

The transepithelial electrical resistance (TEER) of Calu-3, SPOC1 and UNCN3T (cultured on collagen IV-coated Transwell-Clear™ inserts) cells was measured over ~ 30 days, as an indicator of operational tight junctions, and before and after each transport experiment. Measurements were acquired using an EVOM voltohmmeter in conjunction with silver ‘chopsticks’ electrodes (World Precision Instruments Inc., Sarasota, FL). Prior to measurement, pre-warmed transport buffer was added to the cells, 1 mL to the apical chamber and 2 mL to the basolateral chamber. The cells were allowed to equilibrate to 37 °C for 30 min and three measurements were taken for each insert. To reduce variability, chopsticks were held in the same position in each well, taking care not to touch the membrane or insert. An average value was calculated (T_test_) per insert from which the average of an unseeded control (T_control_) was subtracted, to derive the TEER of the cell layer (T_cells_):

$$T_{cells}=\left(T_{test}-T_{control}\right)\times 1.12\left(area\;of\;membrane,\;cm^{2}\right)$$

The resistance of blank or Geltrex™-coated inserts generally did not exceed 100 Ω.cm^2^. The TEER (Ω.cm^2^) of SPOC1 cells cultured on both Transwell-COL™ inserts and Transwell-Clear™ inserts coated with Geltrex™ was compared.

### 2.6 Tight junction characterisation of UNCN3T cells

At 21 days post ALI, UNCN3T cells cultured on Geltrex™-coated Transwell-Clear™ inserts were fixed using 10 % neutral buffered formalin mixed 1:1 with BEGM for 10 min, followed by 10 % neutral buffered formalin alone for 10 min. Each insert was then washed gently three times with PBS/0.1 % Tween (PBST). Cells were permeabilised for 10 min in 0.1 % Triton X-100, washed three times with PBST and blocked for 30 min at room temperature in PBST/1 % gelatin. Antibodies were diluted 1 in 100 in PBST/1 % gelatin and 100 μL was added to each insert drop wise. The antibodies chosen were zonula occludens-1 (ZO-1) (ZO-1 (D7D12) rabbit mAb, 8193), occludin (occludin (E6B4R) rabbit mAb, 91131) (both tight junction proteins) and the adherens junction proteins E-cadherin (E-cadherin (24E10) rabbit mAB, 3195) and β-catenin (β-catenin (D10A8) XP rabbit mAb, 8480) (all from Cell Signaling Technology, Danvers, MA). A control without any primary antibody was included as well as a rabbit IgG1 isotype control (Rabbit (DA1E) mAB IgG XP isotope control, 3900) (Cell Signaling Technology). Primary antibodies were incubated overnight at 2-8 °C in a humidified chamber.

Inserts were gently washed three times in PBST before the addition of anti-rabbit IgG (H + L), F(ab')_2_ fragment (AlexaFluor 488 conjugate, 4412) (Cell Signaling Technology), diluted 1 in 200 in PBST/1 % gelatin, to each insert drop wise as before (100 μL per slide). Inserts were incubated in the humidified chamber at room temperature for 1 h before washing three times in PBST as before. For mounting, the membrane was gently excised using a clean scalpel, laid onto a microscope slide and mounted with a circular coverslip using Prolong Gold Antifade® with DAPI (Invitrogen). Excess mounting medium was blotted using a lens tissue and the slides left to cure at room temperature in the dark. Slides were then imaged using a confocal laser scanning microscope (Leica TCS SP5).

### 2.7 Mucin gene expression

All cell types were cultured to ~ 80 % confluency in the appropriate maintenance medium on 100 mm diameter plates. For UNCN3T cells the plates were coated with 50 μg mL^−1^ collagen 1. RNA was isolated from the cells with Trizol (Invitrogen), according to the manufacturer’s specifications, and resuspended in 100 μL diethyl pyrocarbonate-treated deionised water (Ambion, Thermo Fisher Scientific). Generation of cDNA was achieved using the avian myeloblastosis virus (AMV) reverse transcription kit (Promega, Madison, WI), according to the manufacturer’s instructions, following a DNase treatment step with RNase-free DNase (Promega).

For quantitative PCR (qPCR), total human lung RNA (Applied Biosystems, Thermo Fisher Scientific) was used as a positive control for both human cell lines (Calu-3 and UNCN3T) and total rat lung RNA (AMSBIO, Abingdon, UK) was used for the SPOC1 cell line. The human oral squamous cell carcinoma cell line H376 (European Collection of Authenticated Cell Cultures, Salisbury, UK) was used as a negative control for all cell lines. Primers for SPOC1 cells were obtained as a custom order (IDT Technologies, Leuven, Belgium), with the following sequences; *Muc5ac FOR,* ACT GTT ACT ATG CGA TGT GTA GCC A, *Muc5ac REV,* GAG GAA ACA CAT TGC ACC GA, *Muc5b FOR -* GAA CGC CAT ATT CCC GAC ACT, *Muc5b REV -* GCC CCA GGT GGA GGG ACA TAA, *Gapdh FOR -* CAA CTA CAT GGT CTA CAT GTT C, *Gapdh REV -* CGC CAG TAG ACT CCA CGA C. All reactions were performed using a three-step with melt protocol on a Rotor-Gene Q thermal cycler (Qiagen, Manchester, UK), consisting of a hold step (95 °C, 10 min), followed by 40 cycles of 95 °C for 15 s, 58 °C for 20 s and 72 °C for 20 s. Reaction efficiency was determined using a standard curve in the Rotor-Gene Screenclust HRM software package. Relative quantification of gene target was calculated using the Livak (2^-ΔΔCT^) method [37], normalising to the housekeeper gene *Gapdh* for the SPOC1 cells and *TBP* for Calu-3 and UNCN3T cells.

### 2.8 Enhancement of mucin secretion using AMP-PNP (Secretagogue model)

Cells cultured on inserts were arbitrarily divided into two groups termed ‘low mucus’ and ‘high mucus’. At 16 h prior to treatment, ALI medium was replaced with transport buffer (TB) (DMEM, high glucose, with pyruvate for Calu-3 cells; 1:1 DMEM/Ham’s F12 medium, for SPOC1 cells; LHC basal medium for UNCN3T cells), 500 μL apically and 1500 μL basolaterally. Plates were kept on a shock-absorbing foam pad to prevent inadvertent mechanical stimulation of mucin secretion. The apical surface of each culture was gently washed using 500 μL fresh TB, at −16, −3, −2, −1, −0.5 and −0 h. Washes were stored at 4 °C until mucin secretion could be determined using an ELLA (on the day of treatment). At 10, 20 and 30 min, three further washes were performed and the average mucin content used to establish baseline mucin secretion. Following the wash at 30 min (SPOC1 cells) and 40 min (Calu-3 and UNCN3T cells), adenylyl-imidodiphosphate, tetralithium salt (AMP-PNP) (Roche Applied Science, Sigma Aldrich), a non-hydrolysable analogue of the purinergic stimulator adenosine 5′triphosphate (ATP) was added to the apical surface of cultures allocated to the ‘high mucus’ group, in 500 μL TB. Those cultures allocated to the ‘low mucus’ group had 500 μL TB added to the apical surface. Each AMP-PNP/TB apical solution was changed (“like for like”) at 10-minute intervals. After 60 min, cultures allocated to the ‘low mucus’ group were also treated with AMP-PNP in the same way as treated cells, to ensure that all cells had been capable of enhanced mucin secretion (data not shown). To quantify mucin secretion, 100 μL of each wash was added to a high-binding ELISA plate and subjected to an ELLA.

### 2.9 Depletion of mucus by washing (mucus-depleted (MD) model)

**Fig. 1:**
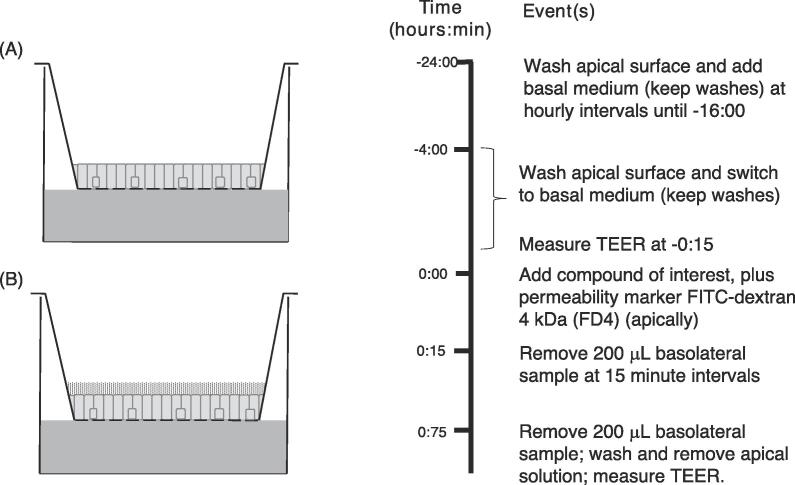
Mucus-depleted (MD) model. Control cultures (A) were washed at hourly intervals to deplete mucus from the apical surface from −24 to −16 h, then again at 30 min intervals between −4 and 0 h. In unwashed cultures (B) mucus was not depleted permitting the effect of mucus on drug transport to be studied.

At 24 h prior to the transport experiment, cultures were arbitrarily assigned to ‘low mucus’ or ‘high mucus’ groups. ‘Low mucus’ cultures were washed apically with the appropriate TB at hourly intervals from minus 24 h to minus 16 h prior to the transport experiment (Fig. 1). ‘High mucus’ cultures were not washed. Cultures were returned to the incubator overnight. At 4 h prior to the transport experiment, medium was aspirated from the basolateral chamber and replaced with TB. Apical washes were resumed at this time point (at 30-minute intervals) and continued until drug application (0 h). All incubations prior to and during transport experiments were performed in a humidified 5 % CO_2_/95 % air environment at 37 °C.

### 2.10 Measurement of testosterone and FD4 transport in the MD model

At t = 0 h, testosterone (2 μM)/FD4 (250 μM) solution (500 μL) was added to the apical chamber of each culture. Samples (200 μL) were taken from the basolateral chamber at 15-minute intervals and replaced with 200 μL TB at each time point, to maintain basolateral volume. At the final time point, a sample (300 μL) was also taken from the apical chamber (Fig. 1). Mucin present in the apical chamber was quantified using the ELLA. Testosterone was quantified using LCMS; each sample (5 μL) was injected into an Agilent™ 1200 binary HPLC system (Agilent Technologies, Santa Clara, CA) coupled to a Bruker HCT Iontrap (Bruker Corporation, Billerica, MA) with electrospray ionisation (ESI). The analyte was monitored in positive ion mode following optimisation via infusion. Separation was achieved using an Ascentis® C18 column, 2.1 mm i.d, 100 mm length, particle size 3 μm (Supelco, Sigma Aldrich), without any prior extraction, using 70% methanol as the mobile phase. Resulting peaks were manually integrated using HyStar™ Acquisition 3.1 software (Bruker Corporation).

FD4 was quantified using fluorescence spectrometry using a fluorimeter (Cary Varian Eclipse, Agilent Technologies, Waldbronn, Germany), with an excitation wavelength of 490 nm and emission wavelength of 520 nm.

The apparent permeability coefficient (P_app_) of testosterone and FD4 was calculated using equation (1):

(1) $$P_{app}=\left(\Delta Q/\Delta t\right)/\left(A.C_{o}\right)$$

Where (ΔQ/Δt) is the amount of testosterone/FD4 transported versus time, A is the area of the Transwell-Clear™ membrane, and C_o_ is the initial concentration of testosterone/FD4 in the donor chamber.

### 2.11 Quantification of mucin secretion using an ELLA

For all cell lines an aliquot (100 μL) of sample was bound in triplicate to a 96-well high-binding microtitre plate (Corning Costar) overnight at 4 °C. Wells were then washed three times with phosphate buffered saline (PBS) containing 0.05 % v/v Tween, 0.5 % w/v bovine gelatin type B (Sigma Aldrich), and blocked by incubation with PBS containing 1.0 % w/v gelatin at 37 °C for 1 h. After further washing, wells with samples from Calu-3 and SPOC1 cells were incubated at 37 °C for 1 h with horseradish peroxidase (HRP)-conjugated *Helix pomatia* agglutinin (HPA-HRP) lectin (EY Laboratories, San Mateo, CA) for Calu-3 cells and horseradish peroxidase-conjugated wheat germ agglutinin (WGA-HRP), isolated from *Triticum vulgaris* (Vector Laboratories, Burlingame, CA) (5.0 μg ml^−1^ in PBS) for SPOC1 cells. Wells with samples from UNCN3T cells were incubated with biotinylated-*Helix pomatia* agglutinin prepared in the blocking solution. These wells were then washed before incubation with HRP-labelled streptavidin. Finally, plates containing samples from all cell lines were washed repeatedly and developed by the addition of substrate solution which consisted of *o*-phenylene diamine (Sigma AldrichFast™ tablet) prepared according to manufacturer’s instructions, for 3 min at room temperature, after which the reaction was terminated by the addition of 20% (v/v) H_2_SO_4_. The absorbance at 492 nm was measured using an Ascent Multiskan™ plate reader (Thermo Fisher Scientific). The absorbance of the samples was compared with known mucin standards, purified by two rounds of density gradient ultracentrifugation, as described by Carlstedt *et al.* [38], from the sputum of a volunteer with chronic obstructive pulmonary disease. Ethical approval was obtained from the University of Brighton School of Pharmacy and Biomolecular Sciences Ethics Committee, under reference PABSREC 0509, and informed consent was obtained from the volunteer.

### 2.12 Quantification of cytokine release and mucus secretion in response to treatment with inhaled drugs

SPOC1 or UNCN3T cells were seeded onto 24-well tissue culture treated plates, pre-coated with a 1/20 dilution of Geltrex™. Cells were seeded at 5 × 10^4^ cells/well and grown to confluence in the appropriate maintenance medium. The cells were washed four times (20 min per wash) with TB appropriate to each cell line after which the cells were incubated for 30 min at 37 °C 5% CO_2_/95% air in control or drug solutions prepared in the appropriate transport buffer. Ipratropium bromide, tiotropium bromide, salbutamol sulfate and budesonide (5-50 μM), were prepared in DMEM/F12 medium (SPOC1 cells) and LHC basal medium (UNCN3T cells). Vanadium (IV) oxide sulfate (VOSO_4_) (20 μg mL^−1^ (123 mM)) (Sigma Aldrich) and AMP-PNP (100 μm) were used as positive control for studies on cytokine release and mucus secretion respectively. Samples from SPOC1 cells were analysed for three rat cytokines; these were IFN-γ, TNF-α and IL-6. Samples from UNCN3T cells (human origin) were analysed for IL-6 and IL-8. Cytokines and mucins were quantified using ELISAs (Legend Max, BioLegend San Diego, CA) according to the manufacturer’s instructions and ELLA respectively.

### 2.13 Statistical analysis

Results are presented as mean and standard deviation. TEER measurements were compared using the unpaired *t*-test when comparing variables (control with treatment) and using the paired *t*-test when comparing the same variable at different time points of secretion and transport experiments (‘before’ and ‘after’). Mucin quantity and values of P_app_ were compared using the non-parametric Mann-Whitney (U) test, since the number of replicates (normally 4) was considered too low to assume a normal distribution. The Kruskal-Wallis test (non-parametric ANOVA) followed by a Dunn’s Multiple Comparisons test was used to assess variability in the permeability of the cells to FD4 across all drug transport experiments and also to compare multiple drug concentrations along with their corresponding controls in the drug irritancy investigations. In all cases, a value of P < 0.05 was considered to be significant.

## 3 Results

### 3.1 A comparison of the TEER of Calu-3, SPOC1 and UNCN3T cells

**Fig. 2:**
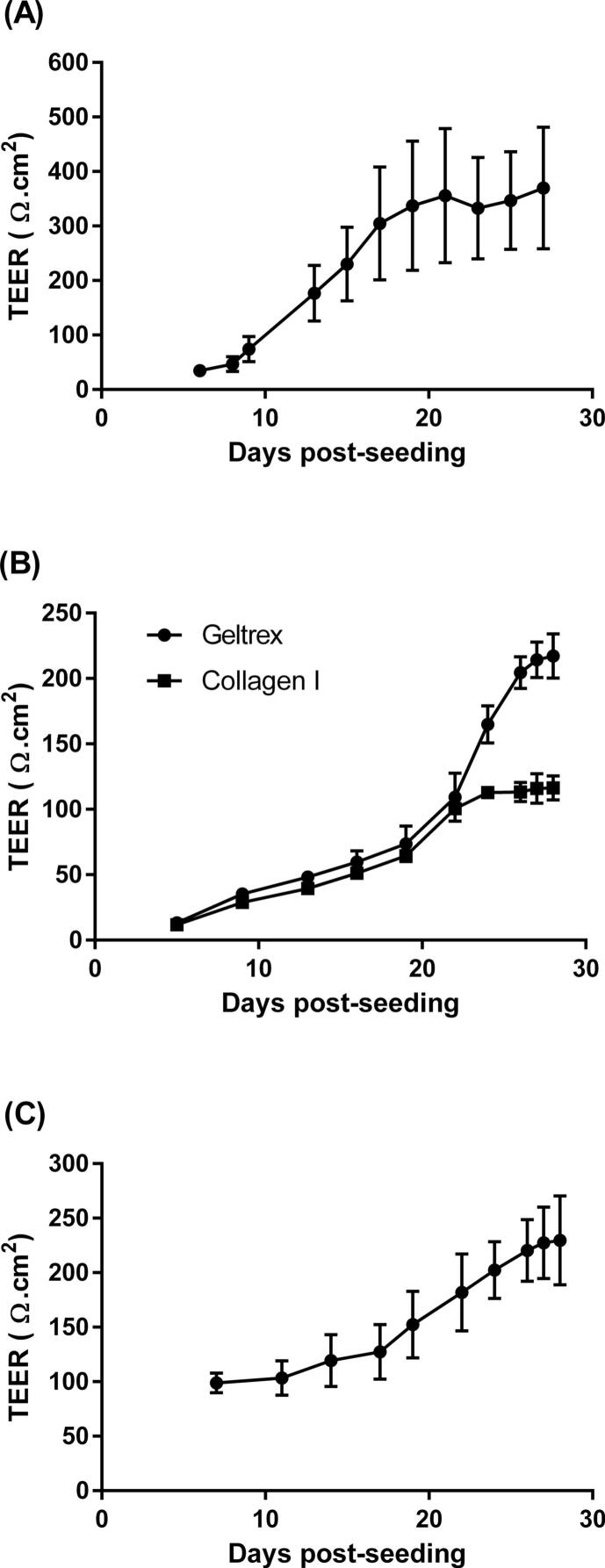
TEER of (A) Calu-3 cells, (B) SPOC1 cells and (C) UNCN3T cells measured over 28 days in culture. Cells were raised to ALI at 7 days post-seeding. SPOC1 cells (B) were seeded onto Transwell-COL and Geltrex™-coated Transwell-CLEAR inserts. Mean ± SD; n = 12 inserts (4 replicates, 3 independent experiments) (Calu-3 cells); n = 16 inserts (4 replicates, 4 independent experiments) (SPOC1 cells, Transwell-COL inserts) and n = 32 inserts (4 replicates, 8 independent experiments) (SPOC1 cells, Geltrex™-coated Transwell-CLEAR inserts); n = 8 inserts (4 replicates, 2 independent experiments) (UNCN3T cells, Collagen-coated Transwell-CLEAR inserts).

The TEER of Calu-3 cells cultured on uncoated Transwell-Clear™ inserts was monitored over 28 days achieving an average TEER of 368 ± 183 Ω.cm^2^ by 21 days of ALI culture conditions (Fig. 2A). In previous studies, SPOC1 cells have been cultured on Transwell-COL™ inserts [19], [21]. However, in the current study, it was found that coating Transwell-Clear™ inserts with a 1/20 dilution of Geltrex™ increased the TEER, from an average of 116 ± 5 (Transwell-COL™ inserts) to 217 ± 18 Ω.cm^2^ after 21 days of ALI culture (28 days post-seeding) (Fig. 2B), an increase of 87 % (unpaired *t*-test, P < 0.001; n = 4). UNCN3T cells cultured on collagen IV-coated Transwell-Clear™ inserts achieved a mean TEER of 229 ± 20 Ω.cm^2^ at 21 days of ALI culture (28 days post-seeding) (Fig. 2C), with values above 100 Ω.cm^2^ being measured ≥ day 11 post seeding.

This was not improved by culturing the UNCN3T cells on Geltrex™-coated inserts which gave a TEER of 196 ± 3 Ω.cm^2^ (unpaired *t*-test, P > 0.05; n = 8).

### 3.2 Tight junction characterisation in UNCN3T cells by immunofluorescence

**Fig. 3:**
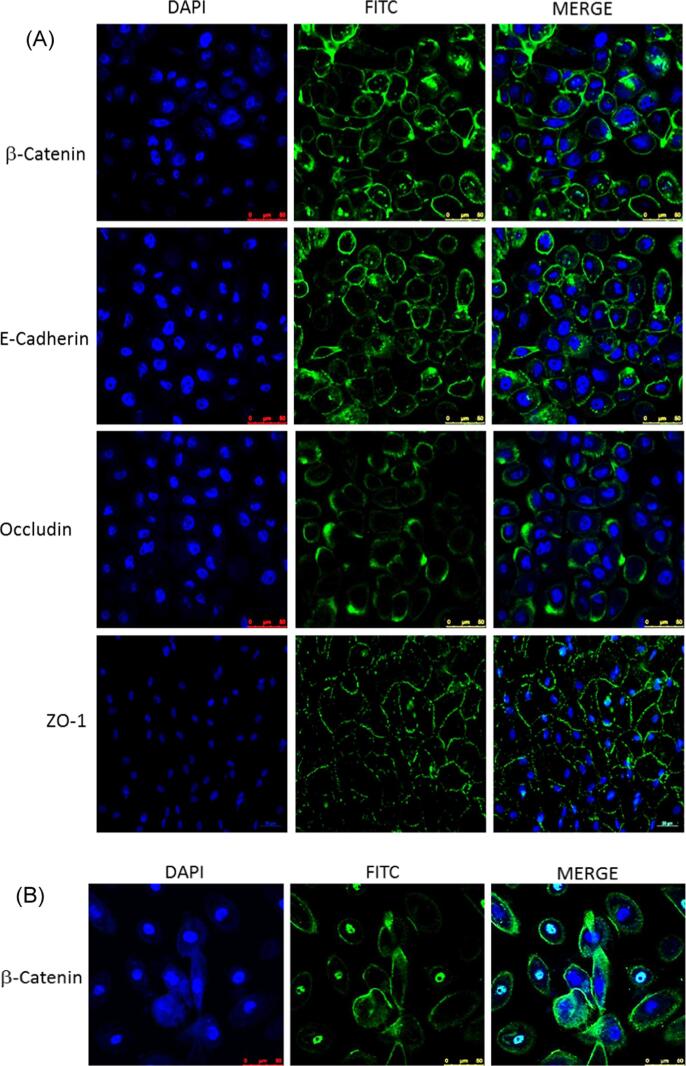
Confocal images of immunostained UNCN3T cells showing clear evidence of (A) tight junctions (ZO-1 and occludin) and adherens junctions (E-cadherin and the E-cadherin-associated protein, β-catenin) after 28 days in culture. (B) After seven days in culture, nuclear β-catenin was observed. Left hand panels show the cell nuclei. The central panels show the target protein and the right hand panels show the two images when merged. Scale bar 50 μm.

After 28 days in culture (21 days at ALI), the adherens junction proteins, β-catenin and E-cadherin (Fig. 3A) could be observed at the cell membranes. Both the tight junction proteins occludin and ZO-1 also stained strongest at the periphery of the cells. Nuclear β-catenin, an indicator of proliferation [39], was observed in cells after seven days of culture; observations were made at seven days to maximize staining for proliferating cells, which would be reduced in a differentiated culture following 21 days at ALI (Fig. 3B).

### 3.3 Mucin expression in Calu-3, SPOC1 and UNCN3T cells

The expression of *MUC5AC* and *MUC5B* was found to be higher in both human cell lines (Calu-3 and UNCN3T) relative to human lung total RNA and normalised to TATA-binding protein (*TBP*) expression. Although, the expression of *MUC5B* was less than the expression of *MUC5AC*. The negative control cell line H376 expressed negligible quantities of *MUC5AC* and *MUC5B*. A similar profile was observed for the expression of *Muc5ac* and *Muc5b* in the rat cell line (SPOC1) compared to rat lung total RNA and normalised to *Gapdh*, whilst the negative control cell line H376 expressed negligible quantities of *Muc5ac* and *Muc5b* mRNA. This indicates a goblet cell phenotype in all cultures (Table 1).

**Table 1:** Expression of *MUC5AC/ Muc5ac* and *MUC5B/Muc5b* in Calu-3, SPOC1 and UNCN3T cells relative to total lung RNA (positive control) and H376 cells (negative control). * Human cell lines (Calu-3 and UNCN3T) and ** Rat cell line (SPOC1).

Cell line	Expression relative to total lung RNA	
	MUC5AC*/Muc5ac**	MUC5B*/Muc5b**
Calu-3	175	33
SPOC1	95	41
UNCN3T	154	41
H376	Negligible (0.01–0.22)	Negligible (0.08, 0.13)

### 3.4 Mucin secretion: A comparison of secretagogue and mucus-depleted models for each cell line

It is well established that extracellular ATP is a potent stimulus for regulated mucus secretion via purinergic receptors [24], [25], [26]. Therefore, cells treated with AMP-PNP, a non-hydrolysable analogue of ATP, were expected to secrete more mucus than untreated cells. However, variable results were obtained from the three cell lines with Calu-3 cells unable to respond to the secretagogue while SPOC1 and UNCN3T cells were responsive.

#### 3.4.1 Calu-3 cells

**Fig. 4:**
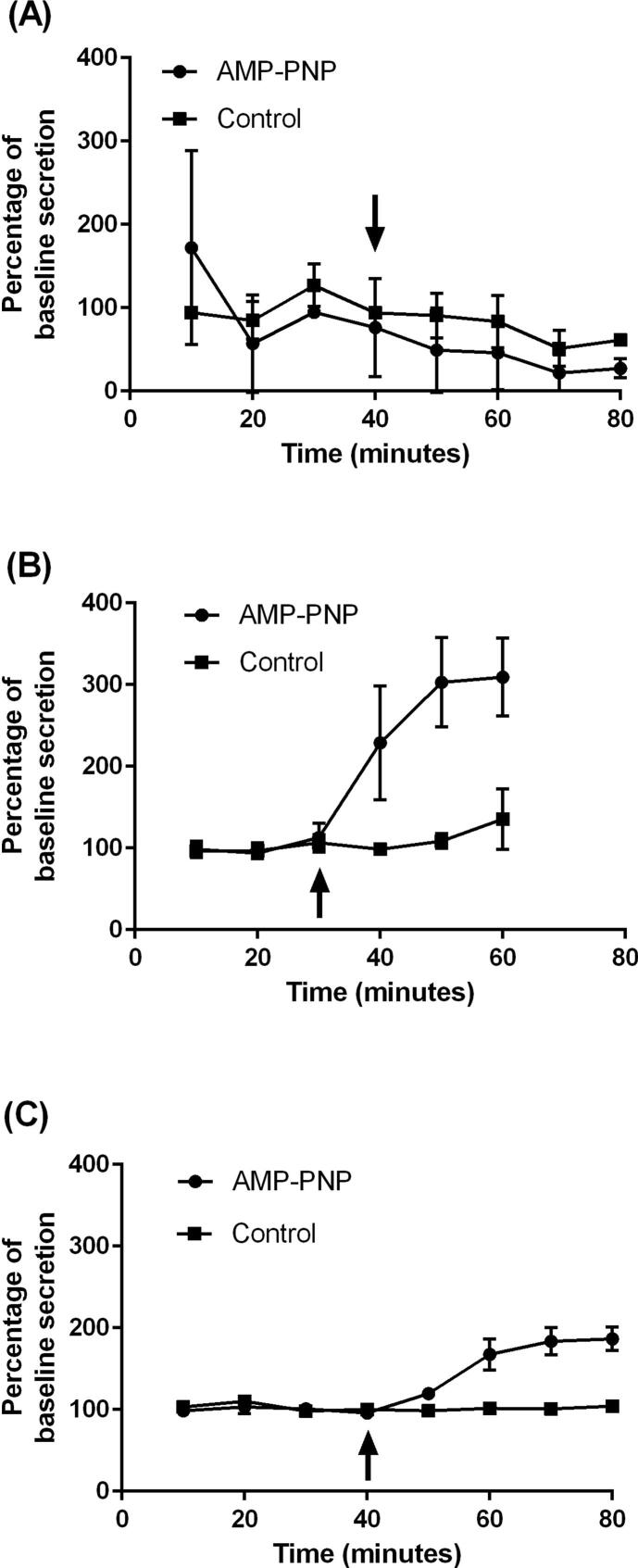
The effect of AMP-PNP (100 μM) on mucin secretion by (A) Calu-3 cells, (B) SPOC1 cells and (C) UNCN3T cells. Mean ± SD; n = 6 inserts (3 replicates, 2 independent experiments) (Calu-3 cells); n = 4 inserts (4 replicates, 1 experiment) SPOC1 and UNCN3T cells). Arrows indicate time of application of AMP-PNP.

**Fig. 5:**
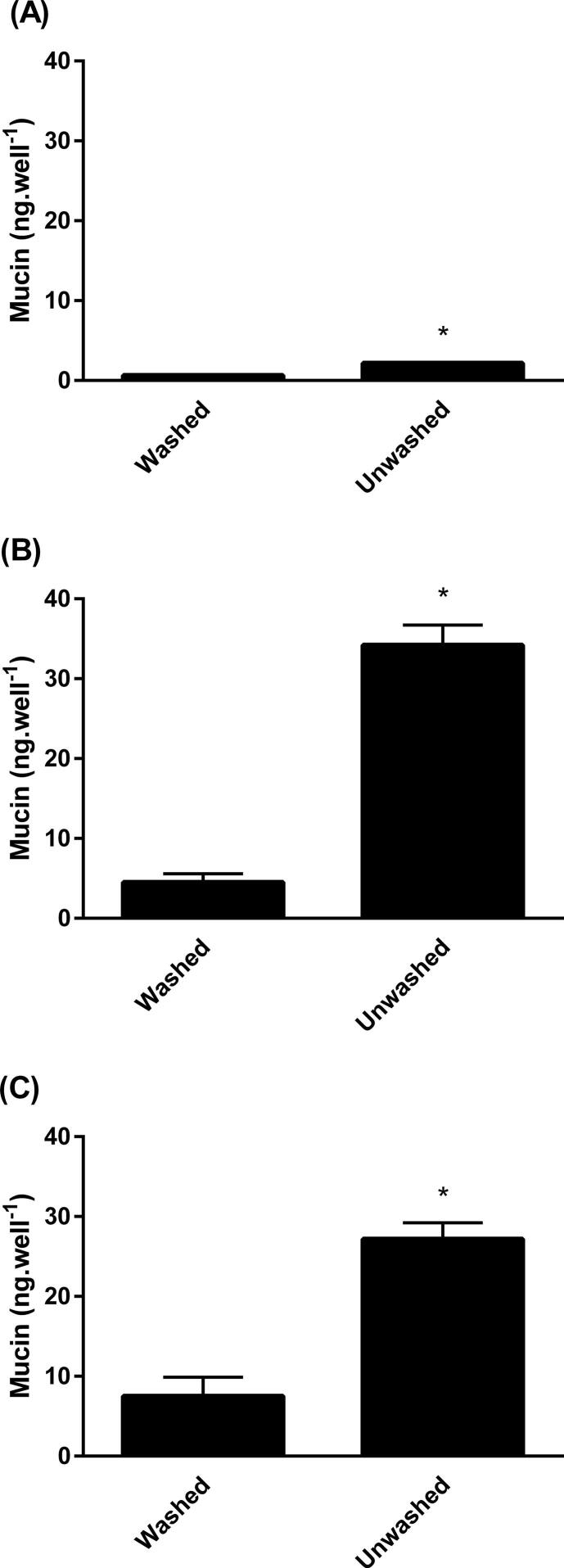
A comparison of the mucin content of the apical chamber at the end of a transport experiment in washed (mucus-depleted) and unwashed (A) Calu-3, (B) SPOC1 and (C) UNCN3T cells. Mean ± SD; n = 4 inserts (4 replicates, 1 independent experiment); n = 8 inserts (4 replicates, 2 independent experiments) for UNCN3T cells. *P < 0.05.

The results indicated that while the ELLA was capable of detecting mucin from untreated and treated Calu-3 cells (lower limit of quantification calculated to be 0.40 ng/well), no significant increase was observed in the quantity of mucin secreted upon application of AMP-PNP (P > 0.05) (Fig. 4A). Later treatment of the control wells with 100 μM AMP-PNP, usually used to show that cultures are capable of responding to AMP-PNP, also failed to enhance mucin secretion (data not shown). The data displayed variability in the concentration of mucin detected at each time point (as demonstrated by large standard deviation bars). This was attributed to the low quantity of mucin secreted by these cultures, an average of 1.81 ± 0.2 ng/well at baseline. For this reason, samples taken at different time points were compared with baseline levels from the same well, established before treatment. Since Calu-3 cells were unresponsive to AMP-PNP it was not possible to create ‘high mucus’ cultures of Calu-3 cells by the secretagogue method with which to study the permeation of testosterone. Therefore, an alternative approach was taken where ‘low mucus’ cultures were produced by washing the cells (MD model). With this approach the mucin concentration in ‘low mucus’ wells was 0.67 ± 0.08 ng/well and 2.18 ± 0.09 ng/well in ‘high mucus’ wells (P < 0.05) yielding a 3.3-fold difference (Fig. 5A). Thus the MD model permitted Calu-3 cells to be used to study the permeation of testosterone.

#### 3.4.2 SPOC1 cells

Addition of the secretagogue AMP-PNP to SPOC1 cells increased mucin levels 3.0-fold when the cells were cultured on Transwell-COL™ inserts (from a baseline of 1.03 ± 0.16 ng/well to 3.09 ± 0.48 ng/well) (P < 0.05) (data not shown). When SPOC1 cells were cultured on Transwell-Clear™ inserts coated with Geltrex™, AMP-PNP increased mucin levels 3.5-fold (from a baseline of 1.43 ± 0.33 ng/well to 5.01 ± 0.40 ng/well) (P < 0.05) (Fig. 4B).

However, the greatest difference between ‘low’ and ‘high’ mucus cultures resulted from the depletion of mucus by washing (MD model). This resulted in mucin levels of 4.5 ± 1.1 ng/well in ‘low mucus’ wells. In ‘high mucus’ wells the concentration of mucin was 34.2 ± 2.5 ng/well, some 7.6-fold greater (n = 40; P < 0.05) (Fig. 5B).

#### 3.4.3 UNCN3T cells

AMP-PNP significantly increased mucin secretion 2-fold in UNCN3T cells (P < 0.05) from a baseline of 2.91 ± 0.92 ng/well to 5.44 ± 1.63 ng/well after treatment. No increase was seen in the control wells at the same time point (Fig. 4C). However, when control wells were treated with 100 μM AMP-PNP at 100 min, mucin secretion significantly increased 1.5-fold by 120 min (P < 0.05), representing an increase from 2.90 ± ng/well to 4.34 ± 0.83 ng/well (data not shown). Similar to SPOC1 cells, AMP-PNP (100 μM) had no discernible effect upon the TEER of UNCN3T cells (P > 0.05). The mean TEER of wells before treatment with AMP-PNP was 229 ± 20 Ω.cm^2^, decreasing to 219 ± 19 Ω.cm^2^.

The difference in mucin concentration between ‘low’ and ‘high’ mucus UNCN3T cultures could be further increased by depleting mucus from the cultures by washing. The mucin concentration of ‘high mucus’ cultures was 3.6-fold that of ‘low mucus’ cultures (compared to 2-fold with AMP-PNP) (Fig. 5C). The mucin levels were quantified at an average of 7.5 ± 2.4 ng/well in ‘low mucus’ cultures 27.2 ± 2.0 ng/well in ‘high mucus’ cultures (P < 0.05).

### 3.5 The effect of mucus on the permeability of each cell line to FD4 using the MD model

#### 3.5.1 Calu-3 cells

**Fig. 6:**
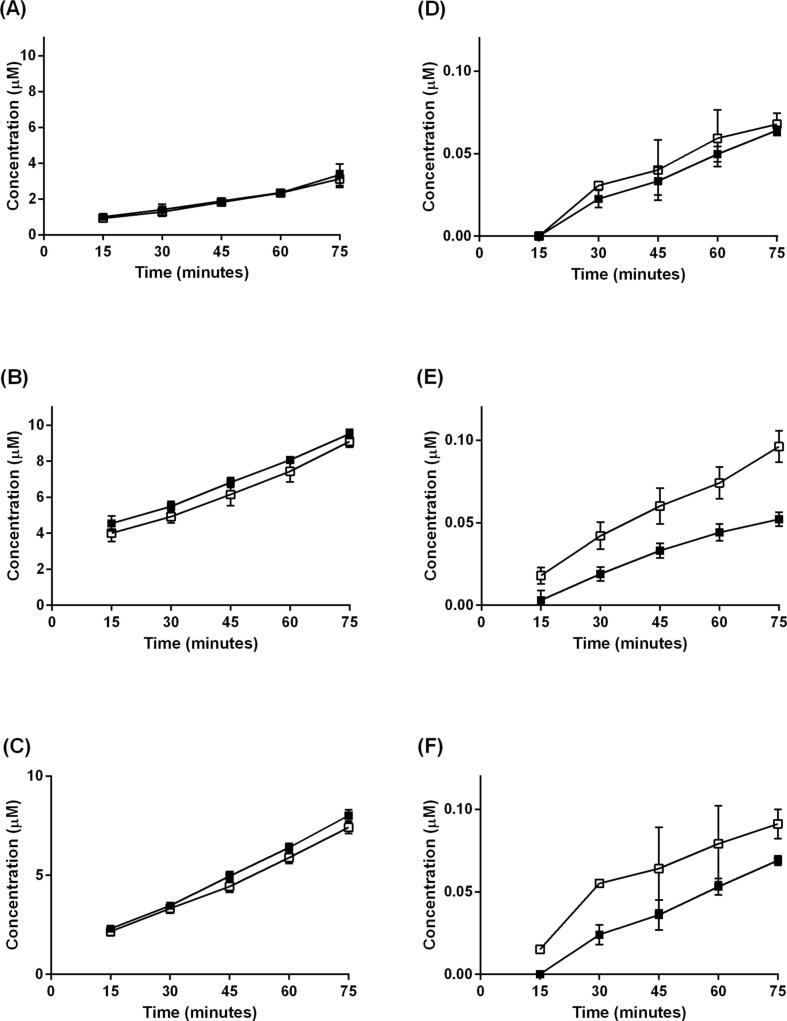
The permeability of washed (mucus-depleted) and unwashed (A) Calu-3, (B) SPOC1 and (C) UNCN3T cells to FD4 and testosterone ((D) Calu-3, (E) SPOC1 and (F) UNCN3T cells). Mean ± SD; n = 4 (4 replicates, 1 independent experiment).

The P_app_ of FD4 in ‘low mucus’ and ‘high mucus’ cultures, was studied concurrently with testosterone (Table 2). It was determined that no significant difference existed between the permeability of Calu-3 cells to FD4 across ‘low mucus’ and ‘high mucus’ cultures with P_app_ values of 3.41 ± 0.22 × 10^-6^ cm.s^−1^ and 3.52 ± 0.35 × 10^-6^ cm.s^−1^ respectively (P > 0.05) (Fig. 6A). TEER was determined to be an average of 462 ± 22 Ω.cm^2^ prior to the transport experiment, falling to 451 ± 39 Ω.cm^2^ (n = 4 inserts). The two sets of values were not significantly different (P > 0.05).

**Table 2:** P_app_ values for the apical to basolateral transport of FD4 and testosterone across washed (low mucus) and unwashed (high mucus) Calu-3, SPOC1 and UNCN3T cultures. The fold difference in mucin concentration and the pre-experimental TEER values are also given. Mean ± SD; n = 4 inserts *n = 40 inserts; **P < 0.05.

			**FD4**		**Testosterone**	
Cell line	Fold difference in mucin	TEER Ω.cm^2^	P_app_ × 10^6^ cm.s^−1^ (low mucus)	P_app_ × 10^6^ cm.s^−1^ (high mucus)	P_app_ × 10^6^ cm.s^−1^ (low mucus)	P_app_ × 10^6^ cm.s^−1^ (high mucus)
**Calu-3**	3.3	462 ± 22	3.41 ± 0.22	3.52 ± 0.35	12.72 ± 2.16	12.19 ± 0.56
**SPOC1**	7.6	205 ± 17*	9.32 ± 0.82	10.14 ± 0.19	12.02 ± 0.39	6.39 ± 1.14**
**UNCN3T**	3.6	196 ± 3	6.30 ± 0.25	6.92 ± 0.39	13.82 ± 2.34	10.59 ± 0.49**

#### 3.5.2 SPOC1 cells

There was no significant difference in the permeability of SPOC1 cells to FD4 in ‘low’ and ‘high’ mucus cultures (P > 0.05); with P_app_ values of 9.32 ± 0.82 × 10^-6^ cm.s^−1^ and 10.14 ± 0.19 × 10^-6^ cm.s^−1^ respectively (Fig. 6B). TEER was an average of 205 ± 17 Ω.cm^2^ prior to the transport experiment (n = 40 inserts), falling to 201 ± 17 Ω.cm^2^. The two sets of values were not found to be significantly different (P > 0.05).

#### 3.5.3 UNCN3T cells

No significant difference existed between permeability of ‘low mucus’ and ‘high mucus’ UNCN3T cells to FD4 with P_app_ values being 6.30 ± 0.25 × 10^-6^ cm.s^−1^ and 6.92 ± 0.39 × 10^-6^ cm.s^−1^ respectively (P > 0.05) (Fig. 6C). TEER was determined to be an average of 196 ± 3 Ω.cm^2^ prior to the transport study, falling to 184 ± 11 Ω.cm^2^ (n = 4 inserts); these values were not significantly different (P > 0.05).

It was concluded, therefore, that the process of washing and differences in mucus did not affect the permeability of the three cell lines to FD4 or the TEER of the cells. Further, the presence of testosterone did not affect the permeability of the cells to FD4 or TEER either.

### 3.6 The effect of mucus on the permeability of each cell line to testosterone using the MD model

It was found that the permeability of Calu-3 cells to testosterone was similar in both ‘low mucus’ and ‘high mucus’ cultures (P > 0.05) using the MD model; Papp = 12.72 ± 2.16 × 10^-6^ cm.s^−1^ and 12.19 ± 0.56 × 10^-6^ cm.s^−1^ respectively (Fig. 6D; Table 2). Conversely, the permeability of SPOC1 cells to testosterone was found to be significantly less in the presence of high concentrations of mucin decreasing from 12.02 ± 0.39 × 10^-6^ cm.s^−1^ to 6.39 ± 1.14 × 10^-6^ cm.s^−1^ (P < 0.05) (Fig. 6E; Table 2). Similar to SPOC1 cells, the permeability of UNCN3T cells to testosterone was significantly lower in ‘high mucus’ cultures compared to ‘low mucus’ cultures (P < 0.05). In ‘high mucus’ cultures the Papp of testosterone was 10.59 ± 0.49 × 10^-6^ cm.s^−1^ compared to 13.82 ± 2.34 × 10^-6^ cm.s^−1^ (low mucus) (Fig. 6F; Table 2).

### 3.7 The effects of some inhaled drugs on cytokine release from SPOC1 and UNCN3T cells

**Fig. 7:**
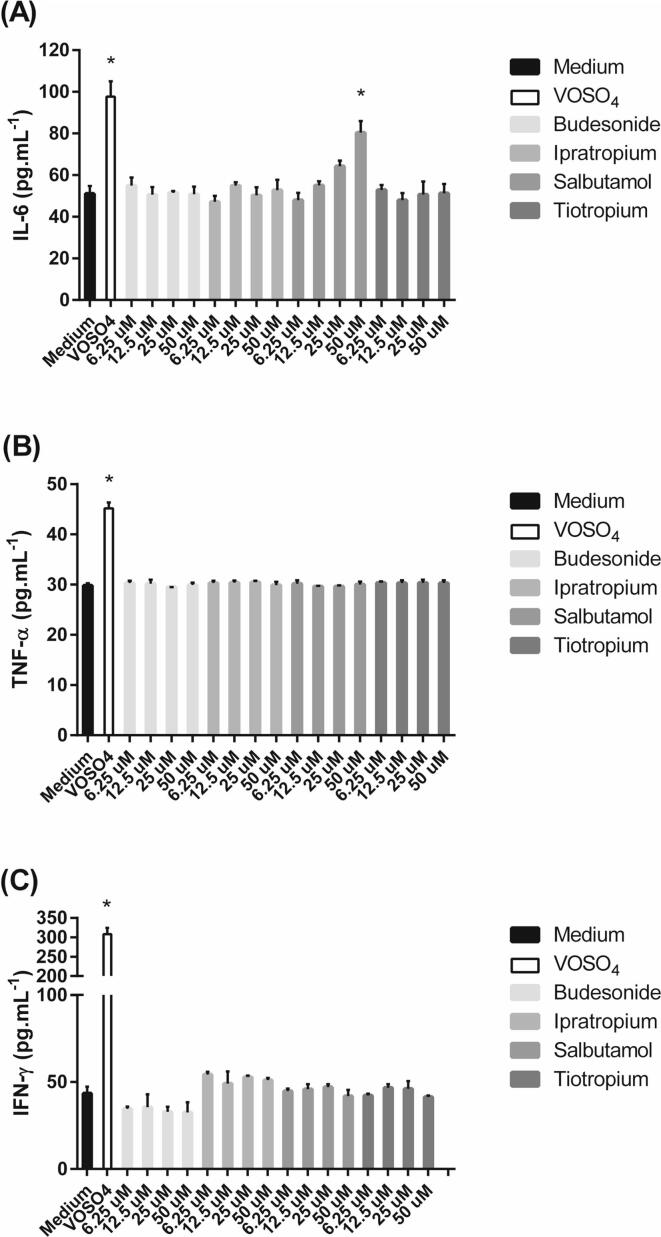
The effect of a number of inhaled drugs on (A) IL-6, (B) TNF-α and (C) INF-γ release from SPOC1 cells cultured on Geltrex™-coated plastic. Vanadium (IV) oxide sulfate (VOSO_4_) (20 μg mL^−1^) was used as a positive control. Mean ± SD; n = 12 (4 replicates; three independent experiments).

Cytokine release was measured in SPOC1 and UNCN3T cells (but not Calu-3 cells) in response to a selection of inhaled drugs. Three rat cytokines (IL-6, TNF-α and IFN-γ) were measured in SPOC1 cells. IL-6 and IL-8 were measured in the human cell line UNCN3T. The treatment of SPOC1 cells with the positive control VOSO_4_ significantly increased IL-6 levels; untreated cells released IL-6 at an average of 51.0 ± 3.7 pg mL^−1^, compared to the positive control of 97.7 ± 7.4 pg mL^−1^ (P < 0.5) (Fig. 7A). No significant increase in IL-6 release was observed in the presence of budesonide, ipratropium or tiotropium at any concentration (P > 0.05). However, salbutamol, caused a dose-dependent increase in IL-6 release from SPOC1 cells, which was significant at 50 μM, (P < 0.05).

The treatment of SPOC1 cells with the positive control VOSO_4_ significantly increased TNF-α levels. Cells exposed to culture medium alone released TNF-α at an average of 29.8 ± 0.5 pg mL^−1^, compared to the positive control of 45.2 ± 1.6 pg mL^−1^ (P < 0.05) (Fig. 7B). No significant increase was observed for budesonide, tiotropium, ipratropium or salbutamol (P > 0.05). The treatment of SPOC1 cells with the positive control VOSO_4_ significantly increased INF-γ levels. Cells exposed to culture medium alone released IFN-γ at an average of 43.4 ± 3.8 pg mL^−1^, compared to the positive control of 308.3 ± 15.9 pg mL^−1^ (P < 0.05) (Fig. 7C). No significant increase was observed for budesonide, tiotropium, ipratropium or salbutamol (P > 0.05).

**Fig. 8:**
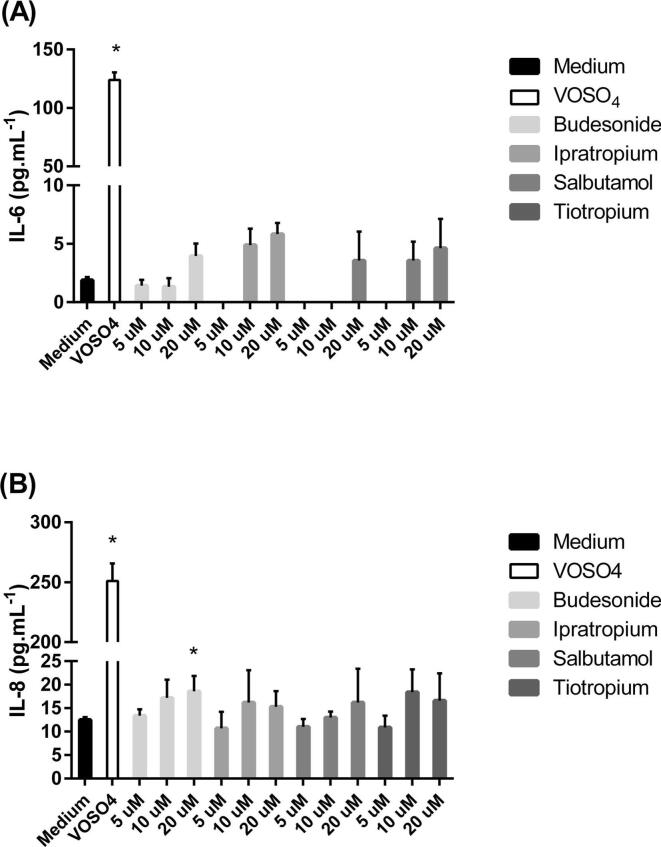
The effect of a number of inhaled drugs on (A) IL-6 and (B) IL-8 release from UNCN3T cells cultured on Geltrex™-coated plastic. Vanadium (IV) oxide sulfate (VOSO_4_) (20 μg mL^−1^) was used as a positive control. Mean ± SD; n = 12 (4 replicates; three independent experiments).

The treatment of UNCN3T cells with the positive control VOSO_4_ increased IL-6 levels from 1.9 ± 0.3 pg mL^−1^ in cells exposed to culture medium alone to 124.0 ± 6.5 pg mL^−1^ in cells exposed to VOSO_4_ (P < 0.05) (Fig. 8A). No significant increase in IL6 was observed for any of the applied drugs (P > 0.05). The treatment of UNCN3T cells with the positive control VOSO_4_ also increased IL-8 levels. Cells exposed to culture medium released IL-8 at an average of 12.5 ± 0.6 pg mL^−1^, compared to the positive control of 251.1 ± 14.5 pg mL^−1^ to 200-fold that of untreated cells (P < 0.05) (Fig. 8B). Only 20 μM budesonide significantly increased the release of IL-8 (P < 0.05).

### 3.8 The effects of some inhaled drugs on mucus secretion from SPOC1 and UNCN3T cells

**Fig. 9:**
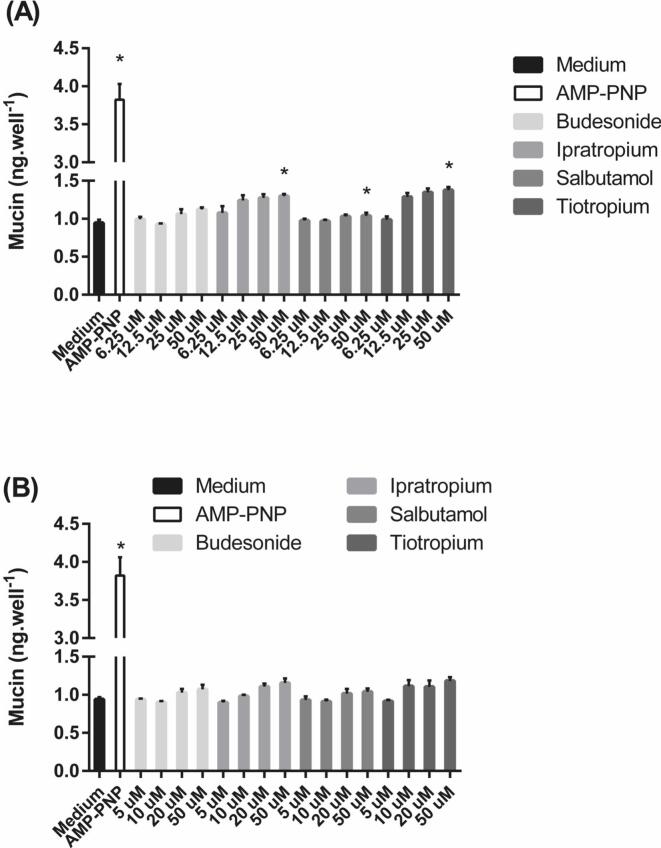
The effect of a number of inhaled drugs on mucin secretion from (A) SPOC1 and (B) UNCN3T cells cultured on Geltrex™-coated plastic. AMP-PNP (100 μm) was used as a positive control. Mean ± SD; n = 12 (n = 4 in triplicate; three experiments performed on different days).

Mucus secretion in SPOC1 and UNCN3T cells (but not Calu-3 cells) was measured in response to a selection of inhaled drugs. Treatment of SPOC1 cells with AMP-PNP (100 μM) increased mucin secretion from an average of 0.94 ± 0.04 ng/well to 3.82 ± 0.21 ng/well (Fig. 9A). Salbutamol, ipratropium and tiotropium significantly increased mucin secretion at 50 μM compared to controls (P < 0.05).

Treatment of UNCN3T cells with AMP-PNP (100 μM) increased mucin secretion from 0.94 ± 0.04 ng/well in cells exposed to culture medium alone to 3.22 ± 0.24 ng/well (P < 0.05) (Fig. 9B). Although there was a trend that suggested that increasing concentrations of the applied drugs increased the secretion of mucin, none of the values were significantly different to the response to culture medium alone (P > 0.05).

## 4 Discussion

The functions of the epithelial barrier fall into three categories. These are chemical, physical and immunological [1]. The mucus secreted by bronchial epithelial cells, containing antimicrobial molecules, protease inhibitors and antioxidants, provides the former, whilst tight junctions regulate the macromolecular and ionic permeability and polarity of the epithelial barrier, thus fulfilling the physical role. Lastly, bronchial epithelial cells have the capacity to release cytokines, chemokines and growth factors which work together to constitute the immunological barrier.

Potential barriers to the absorption of inhaled drugs include the mucus layer and the epithelium itself. Therefore the parameters chosen to determine the suitability of the three cell lines for use as models to study the effect of mucus on drug absorption in the nose and lung were: the ability of the cells to form a tight cell layer, as measured by TEER and the paracellular diffusion of the fluorescent marker FD4; mucin gene expression; secretion of mature, glycosylated mucin; the ability of the cells to secrete mucin in response to a physiological secretagogue; and their ability to show a difference in permeability to testosterone in the presence and absence of mucus. The current study is the first of its kind to use SPOC1 and UNCN3T cells as models with which to study the effect of mucus on drug absorption.

It was not an aim of the study to culture all cell types under identical conditions and then compare their utility. In fact, it is unlikely that the same culturing conditions would be optimal for each cell type due to the nature of immortalisation. Whilst Calu-3 and SPOC1 are both oncogenic in nature, being routinely cultured in DMEM-derived media formulations, UNCN3T is not, exhibiting a phenotype closer to its wild type counterpart. Therefore, each cell line was cultured using conditions reported by previous researchers, with some minor modifications, such as the introduction of Geltrex™ as an extracellular matrix for SPOC1 and UNCN3T cells. So while all cells were cultured at ALI, which enhances differentiation [40], SPOC1 and UNCN3T cells were seeded onto coated inserts while Calu-3 cells were not, and different media were employed for each cell type. It is acknowledged that differences in culturing method could have an impact on cell differentiation, mucin expression and secretion and drug permeation. However, culturing each cell type under the same conditions could also impact these.

### 4.1 Barrier function of Calu-3, SPOC1 and UNCN3T cells

A primary requirement of a cell culture model of the airway epithelium is that it should provide a barrier similar to the native epithelium, particularly if it is intended to be predictive in its behaviour. Other important criteria to consider in the choice of model to study drug permeation include the presence of relevant receptors, evidence of efflux and uptake membrane transporters and metabolic enzymes. Although these were not investigated in the current study.

The TEER of well-differentiated primary cultures of human airway epithelia can exceed 800 Ω.cm^2^ [41]. Perhaps surprisingly, this does not reflect the human airway epithelium *in vivo* which is reported to be moderately leaky; excised bronchial and nasal tissues displaying a relatively low TEER, ≤ 100 Ω.cm^2^ [42], [43]. The TEER of the SPOC1 and UNCN3T cultures is closer to these values, and is in agreement with those reported previously [23]. This supports the use of these cell lines as drug absorption models of the airways as they will better predict the absorption of drugs using the paracellular route. Calu-3 cells cultured at ALI generated TEER values of an average of 368 ± 183 Ω.cm^2^, which agreed with previous studies in which relatively high TEER values were reported [29], [44], [45]. This is greater than is observed in the native tissue and, therefore, would be expected to limit the predictive ability of Calu-3 cells for those compounds absorbed paracellularly. This is supported by a previous study where the P_app_ of fluorescein sodium was shown to remain constant in Calu-3 cells once a TEER of 450 Ω.cm^2^ was achieved, while at TEER values lower than this the paracellular permeability of the cells varied with TEER [46]. It also parallels what is observed with the Caco-2 model of the intestine; the model is tighter than the native epithelium and does not model paracellular absorption as effectively as it models transcellular absorption [47].

Unlike the SPOC1 and Calu-3 cell lines, UNCN3T cells have previously been monitored for cell layer integrity using electrophysiological measurements only with no reference being made to the presence of the tight junction (TJ) proteins. Since TJs are influential in paracellular transport [48] two TJ proteins (ZO-1 and occludin) and one adherens junction (AJ) protein (E-cadherin), together with the AJ associated protein β-catenin were studied. Similar immunofluorescence studies have been undertaken in Calu-3 cells [49].

Confocal images of immunostained UNCN3T cells at 28 days showed evidence of tight junctions and adherens junctions, something not previously reported in these cells. The tight junction proteins, occludin and ZO-1, were located around the edge of the cell membrane, with occludin staining being strongest at the periphery of the cells. This is indicative of the large –COOH domain found in the cytoplasm, which is known to interact with ZO-1 [50]. E-cadherin, the adherens junction protein, was also stained, characteristic of differentiated airway epithelial cells [51] and consistent with the antibody epitope being in the transmembrane region of the protein. β-catenin was located in the membrane region of each cell. This is also indicative of terminally differentiated cells, in which the protein serves to stabilise E-cadherin at the adherens junction. These findings are similar to those observed in primary airway cells cultured at ALI [52], [53] where cells form multilayers. The expression of the junctional proteins is less than might be expected from studies of a single cell type cultured on glass, where the cells are likely to form monolayers, but is consistent with the TEER of the cells. The expression could be explained by the cell composition of the UNCN3T model, which includes the presence of goblet cells. An ultrastructural study showed the TJs around nasal goblet cells to be discontinuous and fragmented [54]. In addition, TJs between enterocytes and goblet cells have been shown to be leakier than between enterocytes [55] and increasing the proportion of goblet cells in cell culture models of the intestine serves to decrease their barrier function [56], [57], [58]. Proliferating cells will also stain for nuclear β-catenin, a result of the Wnt canonical pathway [59]. To assess the cultures for proliferation, an insert was fixed early in the culturing period (at seven days) and probed for β-catenin, in which nuclear staining was clearly seen.

### 4.2 Mucus characterisation of Calu-3, SPOC1 and UNCN3T cells

#### 4.2.1 Gene expression

Characterisation of gene expression used qRT-PCR, normalising to human (Calu-3, UNCN3T) or rat (SPOC1) total lung RNA, showed that both *MUC5AC* and *MUC5B* were expressed in the Calu-3 cell line, with relative amounts that were in agreement with the literature [29]. *Muc5ac and Muc5b* were expressed in the SPOC1 cell line, a finding not previously reported, since previous characterisation used the RTE11 monoclonal antibody to detect the apomucin [21] and ELLA to detect the glycosylated mucin [20], [60]. Similarly, previous studies of the UNCN3T cell line had revealed a mucous cell population [23], but did not include qRT-PCR studies of gene expression or characterisation of mucin secretion. The current study found that both *MUC5AC* and *MUC5B* were expressed by UNCN3T cells (using qRT-PCR). Thus, all three cell lines were found to express the relevant airway mucin genes and the ELLA confirmed the mature, glycosylated status of mucins secreted by the cell lines. This adds physiological relevance to the use of the cells as *in vitro* models of inhaled drug absorption in the presence of mucus.

#### 4.2.2 ‘High’ and ‘low’ mucus models

To study the effect of mucus on drug absorption it is important to have cells with minimal mucus and comparator cells with high levels of mucus. A greater difference between ‘high’ and ‘low’ mucus cultures increases the window of sensitivity of the model improving the likelihood that an effect of mucus on drug or particulate absorption will be detected.

In the current study, this difference in mucus levels was achieved in two ways: using a secretagogue (the non-hydrolysable analogue of ATP, AMP-PNP) to create a ‘high mucus’ group or by the washing of certain wells (the mucus-depleted or MD model) to create a ‘low mucus’ group. Only the MD model could be used in all three cell lines and was therefore used to study the permeation of testosterone and FD4.

We present here the first study using the UNCN3T and SPOC1 cell lines to explore washing as a mechanism of mucus depletion in drug absorption studies. However, the removal, or depletion, of mucus by agitation with buffer has been reported previously in an enterocyte-like cell line [2]. While there is the potential for these additional manipulations to affect the integrity of the cell layer, this was not reported in a comparison of intestinal permeability models [56]. This was controlled for in the current study by the monitoring of TEER before and after experiments and recording the basolateral accumulation of the paracellular permeability marker FD4 across all cultures. FD4 has been used in previous studies as a paracellular permeability marker [45], [61], [62], [63]. TEER measurements taken following drug transport studies were > 98 % of those recorded prior to drug application to the apical surface. Furthermore, the P_app_ values calculated for the transport of FD4 concurrent with the drug of interest did not differ between the ‘low mucus’ and ‘high mucus’ cultures and remained linear throughout each transport period. The integrity of the mucus-depleted cultures was therefore concluded to remain uncompromised throughout the study. Further, this data indicated that higher levels of mucus did not affect the TEER or paracellular permeability of the cells. The authors can find no reports where mucus alone affects the TEER of cells. There are reports that increasing the secretion of mucus from cells, either by changing culturing conditions [46] or seeding cultures with different proportions of mucus-secreting cells [56] decreases TEER and increases paracellular permeability but this is more likely to be due to the change in cell phenotype than an effect of the mucous secretion.

#### 4.2.3 Mucus secretion

The SPOC1 cell line has previously been reported to respond to the physiological agonists to the P2Y2 receptor, ATP and its non-hydrolysable analogue AMP-PNP [64], a response confirmed by the present study. Culturing SPOC1 cells on Geltrex™-coated Transwell-Clear™ inserts resulted in a 3.5-fold difference in mucin secretion between control cells and cells stimulated with AMP-PNP. During mucin secretion studies, it was found that wells that had been left untouched after seeding for the duration of ALI culture, contained in excess of 30 ng/well mucin, as quantified by the ELLA. Therefore, in an attempt to provide a greater difference between the comparator groups of ‘low mucus’ and ‘high mucus’ cultures, the ‘mucus-depleted’ model was developed where the focus was switched from enhancing mucus secretion to create ‘high mucus’ wells, to depleting it to create ‘low mucus’ wells. Depletion of mucus by washing gave rise to an almost 8-fold difference in mucin content between mucus-depleted (‘low mucus’) wells and non-depleted (‘high mucus’) wells, increasing the sensitivity of the model to drugs which might interact with mucus. The profile of mucin accumulation over the ALI culture period was not studied but would provide additional characterisation of the cells.

There are no previous studies of the enhancement of mucin secretion in UNCN3T cells, therefore the ability of the UNCN3T cell line to respond to the physiological secretagogue AMP-PNP is a finding not previously reported and suggests the presence of the P2Y2 receptor in this cell line. However, only a 2-fold difference between high and low mucus cultures could be achieved. When exploring the MD model, the UNCN3T cell line behaved very similarly to the SPOC1 cells line, in that mucins in non-depleted cultures were quantified at close to 30 ng/well by the ELLA, this value being 5-fold greater than the mucin concentration achieved when secretion was enhanced by treatment with AMP-PNP. More importantly, the difference in mucin levels between high and low mucus cultures in the MD model was approximately 4-fold, compared to 2-fold in the secretagogue model.

Enhanced mucus secretion was not detected from Calu-3 cells in response to AMP-PNP supporting the findings of Kreda *et al*. [29]. This strongly suggests that Calu-3 cells lack the relevant purinergic receptors and therefore the secretagogue model could not be used for these cells. Further, this result suggested that Calu-3 cells would not be suitable for use in studies using mucin secretion as a marker for drug irritancy. Attempts were made to replicate the MD model using the Calu-3 cell line. Although a 3-fold difference in mucin between the high and low mucus cells was observed, the relatively small amounts of mucin in the non-depleted cells (2.14 ± 0.05 ng/well) put the Calu-3 cells at a disadvantage to both UNCN3T and SPOC1 cells in terms of suitability for drug absorption studies in the presence of mucus.

In summary, both the secretagogue method and MD method could be used to achieve ‘low’ and ‘high’ mucus cultures for SPOC1 cells and UNCN3T cells although the MD method provided a larger signal window. The secretagogue method could not be used with the Calu-3 cells and although the MD method seemed effective, this was not supported by the testosterone absorption studies.

#### 4.2.4 The effect of mucus on drug absorption

In order to validate the model for use in drug absorption studies, it was important to study the permeability of the cells to a compound where mucus was expected to limit drug diffusion. Mucus has been reported to provide a significant barrier to the diffusion of testosterone [2], [3], [30] and therefore testosterone was selected as a model compound rather than any inhaled drugs where the effect of a mucus barrier on drug absorption is unknown. It has been proposed that testosterone strongly interacts with mucus because of its lipophilic nature, via hydrophobic interactions. Although retarded by its hydrophobic interactions with mucus, testosterone is still able to diffuse through mucus due to its small molecular size. The permeability of the SPOC1 and UNCN3T cells to the model drug testosterone was significantly reduced by the presence of mucus. A lag phase was observed for SPOC1 cells suggesting that diffusion of the molecule was held up, followed by a phase where diffusion of the molecule was slower than that observed in mucus-depleted conditions. These results support the proposal that testosterone binds to mucus and the findings of other studies [2], [3], [30], [65], [66]. They also provide validity to the use of the SPOC1 and UNCN3T cell culture models in drug transport studies since they are capable of detecting a drug-mucus interaction if one exists. Mucus did not present a barrier to the absorption of tiotropium across the MD models of SPOC1 and UNCN3T cells (data not shown).

The presence of mucus in the non-depleted wells of the Calu-3 MD model was unable to reduce the permeability of the cells to testosterone. This was attributed to the low quantity of mucin secreted by the cell line. The level of mucin in non-depleted wells (2.14 ± 0.05 ng/well) was >3-fold that of the mucus-depleted wells. This exceeds the signal-to- background ratio guidance of screening assays [67]; however the signal window here was less than a third of that observed in both SPOC1 and UNCN3T cell lines, in which the permeability of the cells to testosterone was reduced significantly across non-depleted cultures.

In a previous study investigating the impingement of aerosolised microparticles on Calu-3 cells, an increase in the transport of the paracellular marker, sodium fluorescein, was observed in Calu-3 cells cultured under liquid which did not form a mucous layer. The presence of mucus on Calu-3 monolayers cultured at ALI was shown to prevent this increase. It was suggested that the mucus was preventing the microparticles damaging the cells rather than it affecting the permeation of the marker [68]. In the current study, the Calu-3 MD model was unable to detect any change in the transport of testosterone, therefore it could not be reliably used to detect changes in the transport of other compounds. This lack of sensitivity coupled with the inability of Calu-3 cells to secrete mucin in response to the application of a physiological secretagogue meant that the SPOC1 and the UNCN3T cell lines appeared to have more potential as models to study the effect of mucus on drug transport. Furthermore, the relatively high TEER values reported for Calu-3 cultures, in addition to those of the current study, place into doubt the use of this cell line as a physiologically relevant model of drug absorption in the airway. Indeed, it has been suggested that models generating a more ‘leaky’ phenotype (and therefore lower TEER values) would be of greater benefit, since it has been proposed that the relative leakiness of tight junctions in airway epithelia contribute to normal airway functioning [41]. Other studies have correlated relatively high *in vitro* TEER measurements with poorly differentiated cultures [69], which should be borne in mind when considering the choice of model in permeability studies.

Whilst TEER is a widely accepted technique used to measure tight junction integrity, it is by no means the most accurate. Further studies might consider the use of impedance spectroscopy or freeze fracture transmission electron microscopy to study TJs in models such as these [70]. However, chopstick electrodes are non-invasive and may be used to monitor live cultures during maturation and differentiation of cell layers.

The current study focuses on the use of these cell lines cultured at ALI on a suspended permeable support. Further studies into the use of these cell lines could include the perfusion of suspended cultures, such as organ-on-a-chip technologies [71], an increasingly common technique and one shown to improve TJ integrity and differentiation of epithelial cell layers [72] for use in pharmacokinetic studies.

#### 4.2.5 The use of *in vitro* models to predict drug irritancy in the airways

A wide range of *in vitro* cell ALI models and assays/endpoints has been reported in studies of irritancy/toxicity to the airways. Models include primary cells e.g. MucilAir™, EpiAirway™ [73], [74] and cell lines e.g. A549, Calu-3 and 16HBE14o- (as reviewed by Hiemstra *et al.* [75]) and endpoints include measurement of TEER, cytokine (IL-6 and IL-8) and mucin secretion, lactate dehydrogenase release and metabolic activity (MTT, resazurin, etc.). The use of SPOC1 and UNCN3T cells has not been reported and the aim of the study was to examine some secretory endpoints for potential use in studies rather than to undertake a full battery of tests.

In addition to the secretion of mucus, bronchial epithelial cells have the capacity to release cytokines, chemokines and growth factors that form the immunological contribution to the epithelial barrier. For the SPOC1 and UNCN3T cell models to be useful in studies of airway toxicity/irritancy it was important to show that they secrete mucus and release cytokines in response to a challenge. Therefore, along with mucus secretion, the cytokine secretory capacity of the cell lines used in the current studies was determined. Calu-3 cells were excluded from these studies as they were unresponsive to the secretagogue AMP-PNP and the amount of mucus quantified in unwashed wells of the MD model was insufficient to act as a barrier to testosterone. Vanadium sulfate (VOSO_4_) is an established positive control for the determination of cytokine response to metal oxides and other nanoparticle inhaled irritants [76] and thus it was used in the current studies, generating a significant increase for all cytokines studied.

IL-6 release by SPOC1 cells in response to the positive control was 1.9-fold that of culture medium alone. While this increase was significant, it fell just below the standard 2-fold signal window required for assay validity [67] suggesting further study to fully validate the use of this end point in irritancy studies with SPOC1 cells. Of the inhaled drugs studied, only the short-acting β₂ adrenergic receptor agonist, salbutamol, at the highest concentration studied (50 μM) increased IL-6 release. This has been reported previously [77], [78] supporting the use of the model. In contrast to the SPOC1 cells, IL-6 release by UNCN3T cells in response to VOSO_4_ was 66-fold that of culture medium, which may be a reflection of increased differentiation potential, which would better represent the *in vivo* phenotype [1]. This is well in excess of the signal window required for assay validity and indicates that IL-6 release would be an appropriate endpoint in studies of toxicity/irritancy in these cells. None of the inhaled drugs significantly increased IL-6 release (concentrations ≤ 20 μM) in agreement with the SPOC1 cells. As reviewed by Rose-John *et al.* [79], IL-6 can be anti-inflammatory via the classic-signaling pathway, which requires the cells to express the IL-6 receptor, or pro-inflammatory via the *trans*-signaling pathway. Cells present in the airways with a high expression of IL-6 receptors include neutrophils, macrophages and some types of T cells (not epithelial cells). Therefore, it is likely that IL-6 release by SPOC1 and UNCN3T cells is pro-inflammatory via the *trans*-signaling pathway in the airway epithelial cells.

Similar to IL-6, the assay signal window for TNF-α release from SPOC1 cells was <2-fold between VOSO_4_ and medium alone, at 1.5-fold. This increase was also significant but once more would suggest that the assay in this instance is not valid. The release of IFN-γ was significantly increased in SPOC1 cells by the positive control (7-fold), but not by any of the inhaled drugs. This indicates that the assay was valid but that the drugs studied did not significantly affect IFN-γ release. The last cytokine studied was IL-8. In the current study, treatment with the positive control resulted in a 20-fold increase in IL-8 release from UNCN3T cells, which validated the assay and supported the previous findings of Fulcher et al. [23]. Surprisingly, the inhaled corticosteroid, budesonide (20 μM) increased the release of IL-8. Budesonide has been reported to reduce the concentration of IL-8 in inflamed lungs [80].

In summary, these results provide evidence that the SPOC1 and UNCN3T cell lines may be useful to predict drug irritancy, using the ELISA assay to measure IL-6 (SPOC1 and UNCN3T), IL-8 (UNCN3T) and IFN-γ (SPOC1) release in response to treatment, but not TNF-α (SPOC1).

In considering increased secretion of mucin as an endpoint for toxicity/irritancy, the standard 2-fold signal window was exceeded for both cell lines, indicating the validity of the assay. Budesonide had no effect on the secretion of mucin either cell line. There are no studies on the effect of budesonide on baseline mucin secretion. However, previous studies have shown that budesonide is able to suppress *MUC5AC* gene expression induced by, for example, phorbol-12-myristate-13 acetate (PMA) [81] and TGF-α [82]. Mucin secretion was increased in SPOC1 cells but not UNCN3T cells by the other inhaled drugs at the highest concentration applied. Both ipratropium and tiotropium are inhaled anticholinergic drugs. As with budesonide, there are no studies on the effect of these drugs on baseline mucus secretion although studies exist on their effect on stimulated mucus secretion. For instance, tioptropium inhibits *MUC5AC* expression stimulated by neutrophil elastase but not by IL-13 [83], [84]. For salbutamol, β2-adrenergic receptors (β2-ARs) are expressed in airway epithelial cells [85] and agonists such as salbutamol may increase the expression of the airway mucins via the MAPK signaling pathway and goblet cell hyperplasia [86], [87]. The findings of the current study indicate that the detection of mucin secretion as an endpoint of irritancy studies is feasible in the SPOC1 and UNCN3T cell. *In vitro-in vivo* correlation studies would be required to confirm its predictive potential.

## 5 Conclusions

The Calu-3, SPOC1 and UNCN3T cell lines have been shown to express airway mucin genes and to secrete mature, glycosylated mucins. Whilst Calu-3 cells may be suitable for drug and particulate transport studies independent of mucus secretion, the current study suggests that they are not suitable for studies aimed at ascertaining the effect of mucus on drug and particulate absorption. This is due to the lack of response to a physiological secretagogue meaning that it is not possible to produce ‘high’ and ‘low’ mucus cultures with this method. Further, the low levels of mucus secreted make the MD method ineffective also. Conversely, both SPOC1 and UNCN3T respond to a physiological secretagogue and offer a broader signal window when using the MD model. The permeability of both the SPOC1 and UNCN3T cell lines to testosterone was significantly reduced in the presence of mucus. This indicates that they are able to provide robust and sensitive models to study the effect of mucus on drug absorption and would also be useful to investigate the mucus penetration, cellular uptake and permeation of nanoparticle-based drug delivery systems. Both the SPOC1 and UNCN3T cell lines showed potential to predict drug irritancy using mucin secretion and cytokine release as endpoints. We therefore propose the use of the mucus-depleted model using SPOC1 or UNCN3T cells in further studies of novel inhaled therapeutics in ADME determination.

## Declaration of Competing Interest

The authors declare that they have no known competing financial interests or personal relationships that could have appeared to influence the work reported in this paper.

## References

[1] Blume C., Swindle E.J., Dennison P., Jayasekera N.P., Dudley S., Monk P., Behrendt H., Schmidt-Weber C.B., Holgate S.T., Howarth P.H., Traidl-Hoffmann C., Davies D.E. (2013). Barrier responses of human bronchial epithelial cells to grass pollen exposure. Eur Respir J.

[2] Hagesaether E., Christiansen E., Due-Hansen M.E., Ulven T. (2013). Mucus can change the permeation rank order of drug candidates. Int J Pharm.

[3] Sigurdsson H.H., Kirch J., Lehr C.M. (2013). Mucus as a barrier to lipophilic drugs. Int J Pharm.

[4] Sporty J.L., Horálková L., Ehrhardt C. (2008). In vitro cell culture models for the assessment of pulmonary drug disposition. Expert Opin Drug Metab Toxicol.

[5] Stewart C.E., Torr E.E., Mohd Jamili N.H., Bosquillon C., Sayers I. (2012). Evaluation of differentiated human bronchial epithelial cell culture systems for asthma research. J Allergy (Cairo).

[6] Lock J.Y., Carlson T.L., Carrier R.L. (2018). Mucus models to evaluate the diffusion of drugs and particles. Adv Drug Deliv Rev.

[7] Forbes B., Ehrhardt C. (2005). Human respiratory epithelial cell culture for drug delivery applications. Eur J Pharm Biopharm.

[8] Marshall LJ, Oguejiofor W, Willetts RS, Griffiths HR, Devitt A. Developing accurate models of the human airways. J Pharm Pharmacol 2014. 10.1111/jphp.12340. 25556403

[9] Lechanteur A., das Neves J., Sarmento B. (2018). The role of mucus in cell-based models used to screen mucosal drug delivery. Adv Drug Deliv Rev.

[10] Forbes B., Shah A., Martin G.P., Lansley A.B. (2003). The human bronchial epithelial cell line 16HBE14o- as a model system of the airways for studying drug transport. Int J Pharm.

[11] Florea B.I., Cassara M.L., Junginger H.E., Borchard G. (2003). Drug transport and metabolism characteristics of the human airway epithelial cell line Calu-3. J Control Release.

[12] McDougall C.M., Blaylock M.G., Douglas J.G., Brooker R.J., Helms P.J., Walsh G.M. (2008). Nasal epithelial cells as surrogates for bronchial epithelial cells in airway inflammation studies. Am J Respir Cell Mol Biol.

[13] Murgia X., Yasar H., Carvalho-Wodarz C., Loretz B., Gordon S., Schwarzkopf K., Schaefer U., Lehr C.-M. (2017). Modelling the bronchial barrier in pulmonary drug delivery: A human bronchial epithelial cell line supplemented with human tracheal mucus. Eur J Pharm Biopharm.

[14] Economou E.C., Marinelli S., Smith M.C., Routt A.A., Kravets V.V., Chu H.W., Spendier K., Celinski Z.J. (2016). Magnetic Nanodrug Delivery Through the Mucus Layer of Air-Liquid Interface Cultured Primary Normal Human Tracheobronchial Epithelial Cells. Bionanoscience.

[15] Brinks V., Lipinska K., de Jager M., Beumer W., Button B., Livraghi-Butrico A., Henig N., Matthee B. (2019). The Cystic Fibrosis-Like Airway Surface Layer Is not a Significant Barrier for Delivery of Eluforsen to Airway Epithelial Cells. J Aerosol Med Pulm Drug Deliv.

[16] Mura S., Hillaireau H., Nicolas J., Kerdine-Römer S., Le Droumaguet B., Deloménie C., Nicolas V., Pallardy M., Tsapis N., Fattal E. (2011). Biodegradable nanoparticles meet the bronchial airway barrier: how surface properties affect their interaction with mucus and epithelial cells. Biomacromolecules.

[17] Meindl C., Stranzinger S., Dzidic N., Salar-Behzadi S., Mohr S., Zimmer A., Fröhlich E., Ahmad S. (2015). Permeation of Therapeutic Drugs in Different Formulations across the Airway Epithelium In Vitro. PLoS ONE.

[18] Cingolani E., Alqahtani S., Sadler R.C., Prime D., Stolnik S., Bosquillon C. (2019). In vitro investigation on the impact of airway mucus on drug dissolution and absorption at the air-epithelium interface in the lungs. Eur J Pharm Biopharm.

[19] Doherty M.M., Liu J., Randell S.H., Carter C.A., Davis C.W., Nettesheim P., Ferriola P.C. (1995). Phenotype and differentiation potential of a novel rat tracheal epithelial cell line. Am. J. Respir. Cell Mol. Biol..

[20] Abdullah L., Davis S.W., Burch L., Yamauchi M., Randell S.H., Nettesheim P., Davis C.W. (1996). P-2u purinoceptor regulation of mucin secretion in SPOC1 cells, a goblet cell line from the airways. Biochem. J.

[21] Randell S.H., Liu J.Y., Ferriola P.C., Kaartinen L., Doherty M.M., Davis C.W., Nettesheim P. (1996). Mucin production by SPOC1 cells - An immortalized rat tracheal epithelial cell line. Am. J. Respir. Cell Mol. Biol..

[22] Abdullah L.H., Bundy J.T., Ehre C., Davis C.W. (2003). Mucin secretion and PKC isoforms in SPOC1 goblet cells: differential activation by purinergic agonist and PMA. Am J Physiol Lung Cell Mol Physiol.

[23] Fulcher M.L., Gabriel S.E., Olsen J.C., Tatreau J.R., Gentzsch M., Livanos E., Saavedra M.T., Salmon P., Randell S.H. (2009). Novel human bronchial epithelial cell lines for cystic fibrosis research. Am J Physiol Lung Cell Mol Physiol.

[24] Lazarowski E.R., Boucher R.C. (2009). Purinergic receptors in airway epithelia. Curr Opin Pharmacol.

[25] Okada SF, Zhang L, Kreda SM, Abdullah LH, William Davis C, Pickles RJ, Lazarowski ER, Boucher RC. Coupled nucleotide and mucin hypersecretion from goblet-cell metaplastic human airway epithelium. Am J Respir Cell Mol Biol 45(2) (2011) 253-60. DOI: 2010-0253OC [pii] 10.1165/rcmb.2010-0253OC. 10.1165/rcmb.2010-0253OC PMC3175555 20935191

[26] Winkelmann V.E., Thompson K.E., Neuland K., Jaramillo A.M., Fois G., Schmidt H., Wittekindt O.H., Han W., Tuvim M.J., Dickey B.F., Dietl P., Frick M. (2019). Inflammation-induced upregulation of P2X4 expression augments mucin secretion in airway epithelia. Am J Physiol Lung Cell Mol Physiol.

[27] Clancy S.M., Yeadon M., Parry J., Yeoman M.S., Adam E.C., Schumacher U., Lethem M.I. (2004). Endothelin-1 inhibits mucin secretion from ovine airway epithelial goblet cells. Am J Respir Cell Mol Biol.

[28] Ehre C., Zhu Y., Abdullah L.H., Olsen J., Nakayama K.I., Nakayama K., Messing R.O., William Davis C. (2007). nPKCepsilon, a P2Y2-R downstream effector in regulated mucin secretion from airway goblet cells. Am J Physiol Cell Physiol.

[29] Kreda S.M. (2007). Coordinated release of nucleotides and mucin from human airway epithelial Calu-3 cells. Journal of Physiology-London.

[30] Khanvilkar K., Donovan M.D., Flanagan D.R. (2001). Drug transfer through mucus. Adv Drug Deliv Rev.

[31] Nie Y.-C., Wu H., Li P.-B., Luo Y.-L., Zhang C.-C., Shen J.-G., Su W.-W. (2012). Characteristic comparison of three rat models induced by cigarette smoke or combined with LPS: to establish a suitable model for study of airway mucus hypersecretion in chronic obstructive pulmonary disease. Pulm Pharmacol Ther.

[32] Dijkstra A.E., Boezen H.M., van den Berge M., Vonk J.M., Hiemstra P.S., Barr R.G., Burkart K.M., Manichaikul A., Pottinger T.D., Silverman E.K., Cho M.H., Crapo J.D., Beaty T.H., Bakke P., Gulsvik A., Lomas D.A., Bossé Y., Nickle D.C., Paré P.D., de Koning H.J., Lammers J.-W., Zanen P., Smolonska J., Wijmenga C., Brandsma C.-A., Groen H.J.M., Postma D.S. (2015). Dissecting the genetics of chronic mucus hypersecretion in smokers with and without COPD. Eur Respir J.

[33] Lenoir J., Claerhout I., Kestelyn P., Klomp A., Remon J.-P., Adriaens E. (2011). The slug mucosal irritation (SMI) assay: development of a screening tool for the evaluation of ocular discomfort caused by shampoos. Toxicol In Vitro.

[34] Lenoir J., Bachert C., Remon J.-P., Adriaens E. (2013). The Slug Mucosal Irritation (SMI) assay: a tool for the evaluation of nasal discomfort. Toxicol In Vitro.

[35] Chand H.S., Woldegiorgis Z., Schwalm K., McDonald J., Tesfaigzi Y. (2012). Acute inflammation induces insulin-like growth factor-1 to mediate Bcl-2 and Muc5ac expression in airway epithelial cells. Am J Respir Cell Mol Biol.

[36] Chung K.F. (2005). Inflammatory mediators in chronic obstructive pulmonary disease. Curr Drug Targets Inflamm Allergy.

[37] Livak K.J., Schmittgen T.D. (2001). Analysis of Relative Gene Expression Data Using Real-Time Quantitative PCR and the 2−ΔΔCT Method. Methods.

[38] Carlstedt I., Lindgren H., Sheehan J.K., Ulmsten U., Wingerup L. (1983). Isolation and characterization of human cervical-mucus glycoproteins. Biochem J.

[39] Ehrlich D., Ehrlich E., Thome M.A., Gutt C.N., von Knebel Doeberitz M., Niggli F., Perantoni A.O., Koesters R. (2010). Nuclear accumulation of beta-catenin protein indicates activation of wnt signaling in chemically induced rat nephroblastomas. Pediatr Dev Pathol.

[40] Yamaya M., Finkbeiner W.E., Chun S.Y., Widdicombe J.H. (1992). Differentiated structure and function of cultures from human tracheal epithelium. Am J Physiol.

[41] Coyne C.B., Gambling T.M., Boucher R.C., Carson J.L., Johnson L.G. (2003). Role of claudin interactions in airway tight junctional permeability. Am J Physiol Lung Cell Mol Physiol.

[42] Knowles M., Murray G., Shallal J., Askin F., Ranga V., Gatzy J., Boucher R. (1984). Bioelectric properties and ion flow across excised human bronchi. J Appl Physiol Respir Environ Exerc Physiol.

[43] Boucher R.C., Stutts M.J., Knowles M.R., Cantley L., Gatzy J.T. (1986). Na+ transport in cystic fibrosis respiratory epithelia. Abnormal basal rate and response to adenylate cyclase activation. J Clin Invest.

[44] Foster K.A., Avery M.L., Yazdanian M., Audus K.L. (2000). Characterization of the Calu-3 cell line as a tool to screen pulmonary drug delivery. Int J Pharm.

[45] Grainger C.I., Greenwell L.L., Lockley D.J., Martin G.P., Forbes B. (2006). Culture of Calu-3 cells at the air interface provides a representative model of the airway epithelial barrier. Pharm. Res..

[46] Ehrhardt C., Fiegel J., Fuchs S., Abu-Dahab R., Schaefer U.F., Hanes J., Lehr C.-M. (2002). Drug absorption by the respiratory mucosa: cell culture models and particulate drug carriers. J Aerosol Med.

[47] Artursson P., Palm K., Luthman K. (2001). Caco-2 monolayers in experimental and theoretical predictions of drug transport. Adv Drug Deliv Rev.

[48] Balda M.S., Whitney J.A., Flores C., González S., Cereijido M., Matter K. (1996). Functional dissociation of paracellular permeability and transepithelial electrical resistance and disruption of the apical-basolateral intramembrane diffusion barrier by expression of a mutant tight junction membrane protein. J Cell Biol.

[49] Wan H., Winton H.L., Soeller C., Stewart G.a., Thompson P.j., Gruenert D.c., Cannell M.B., Garrod D.r., Robinson C. (2000). Tight junction properties of the immortalized human bronchial epithelial cell lines Calu-3 and 16HBE14o. Eur Respir J.

[50] Rao R. (2009). Occludin phosphorylation in regulation of epithelial tight junctions. Ann N Y Acad Sci.

[51] Heijink I.H., Nawijn M.C., Hackett T.-L. (2014). Airway epithelial barrier function regulates the pathogenesis of allergic asthma. Clin Exp Allergy.

[52] Mercier C., Jacqueroux E., He Z., Hodin S., Constant S., Perek N., Boudard D., Delavenne X. (2019). Pharmacological characterization of the 3D MucilAir™ nasal model. Eur J Pharm Biopharm.

[53] Buckley A.G., Looi K., Iosifidis T., Ling K.-M., Sutanto E.N., Martinovich K.M., Kicic-Starcevich E., Garratt L.W., Shaw N.C., Lannigan F.J., Larcombe A.N., Zosky G., Knight D.A., Rigby P.J., Kicic A., Stick S.M. (2018). Visualisation of Multiple Tight Junctional Complexes in Human Airway Epithelial Cells. Biol Proced Online.

[54] Carson J.L., Collier A.M., Knowles M.R., Boucher R.C. (1985). Ultrastructural characterization of epithelial cell membranes in normal human conducting airway epithelium: A freeze-fracture study. Am J Anat.

[55] Madara J.L., Trier J.S. (1982). Structure and permeability of goblet cell tight junctions in rat small intestine. J Membr Biol.

[56] Hilgendorf C., Spahn-Langguth H., Regårdh C.G., Lipka E., Amidon G.L., Langguth P. (2000). Caco-2 versus Caco-2/HT29-MTX co-cultured cell lines: permeabilities via diffusion, inside- and outside-directed carrier-mediated transport. J Pharm Sci.

[57] Wikman-Larhed A., Artursson P. (1995). Co-cultures of human intestinal goblet (HT29-H) and absorptive (Caco-2) cells for studies of drug and peptide absorption. Eur. J. Pharm. Sci..

[58] Walter E., Janich S., Roessler B.J., Hilfinger J.M., Amidon G.L. (1996). HT29-MTX/Caco-2 cocultures as an in vitro model for the intestinal epithelium: in vitro-in vivo correlation with permeability data from rats and humans. J Pharm Sci.

[59] Koch S. (2017). Extrinsic control of Wnt signaling in the intestine. Differentiation.

[60] Rossi A.H., Sears P.R., Davis C.W. (2004). Ca2+ dependency of 'Ca2+-independent' exocytosis in SPOC1 airway goblet cells. J Physiol.

[61] Strengert M., Knaus U.G. (2011). Analysis of epithelial barrier integrity in polarized lung epithelial cells. Methods Mol Biol.

[62] Leng Y. (2014). Effect of acute, slightly increased intra-abdominal pressure on intestinal permeability and oxidative stress in a rat model. PLoS ONE.

[63] Grainger C.I., Greenwell L.L., Martin G.P., Forbes B. (2009). The permeability of large molecular weight solutes following particle delivery to air-interfaced cells that model the respiratory mucosa. Eur J Pharm Biopharm.

[64] Abdullah L.H., Davis C.W. (2007). Regulation of airway goblet cell mucin secretion by tyrosine phosphorylation signaling pathways. Am J Phys Lung Cell and Mol Phys.

[65] Cone RA. Barrier properties of mucus. Adv Drug Deliv Rev, 61(2) (2009) 75-85 DOI: S0169-409X(08)00259-7 [pii] 10.1016/j.addr.2008.09.008. 10.1016/j.addr.2008.09.008 19135107

[66] Larhed A.W., Artursson P., Björk E. (1998). The influence of intestinal mucus components on the diffusion of drugs. Pharm Res.

[67] Iversen P.W., Eastwood B.J., Sittampalam G.S., Cox K.L. (2006). A comparison of assay performance measures in screening assays: signal window, Z' factor, and assay variability ratio. J Biomol Screen.

[68] Fiegel J., Ehrhardt C., Schaefer U.F., Lehr C.-M., Hanes J. (2003). Large porous particle impingement on lung epithelial cell monolayers - Toward improved particle characterization in the lung. Pharm. Res..

[69] Leung C., Wadsworth S.J., Yang S.J., Dorscheid D.R. (2020). Structural and functional variations in human bronchial epithelial cells cultured in air-liquid interface using different growth media. American Journal of Physiology-Lung Cellular and Molecular Physiology.

[70] Srinivasan B., Kolli A.R., Esch M.B., Abaci H.E., Shuler M.L., Hickman J.J. (2015). TEER measurement techniques for in vitro barrier model systems. J Lab Autom.

[71] Barros A.S., Costa A., Sarmento B. (2021). Building three-dimensional lung models for studying pharmacokinetics of inhaled drugs. Adv Drug Deliv Rev.

[72] Yin J, Sunuwar L, Kasendra M, Yu H, Tse C-M, Talbot Jr. C, Boronina TN, Cole RN, Karalis K, Donowitz M. Fluid Shear Stress Enhances Differentiation of Jejunal Human Enteroids in Intestine-Chip. American Journal of Physiology-Gastrointestinal and Liver Physiology. 0(0): p. null DOI: 10.1152/ajpgi.00282.2020. 10.1152/ajpgi.00282.2020 PMC8202237 33074011

[73] Balogh Sivars K, Sivars U, Hornberg E, Zhang H, Brändén L, Bonfante R, Huang S, Constant S, Robinson I, Betts CJ, Åberg PM. A 3D Human Airway Model Enables Prediction of Respiratory Toxicity of Inhaled Drugs In Vitro. Toxicol Sci, 2018. 162(1): p. 301-308 DOI: 10.1093/toxsci/kfx255. 10.1093/toxsci/kfx255 29182718

[74] Jackson G.R., Maione A.G., Klausner M., Hayden P.J. (2018). Prevalidation of an Acute Inhalation Toxicity Test Using the EpiAirway In Vitro Human Airway Model. Appl In Vitro Toxicol.

[75] Hiemstra P.S., Grootaers G., van der Does A.M., Krul C.A.M., Kooter I.M. (2018). Human lung epithelial cell cultures for analysis of inhaled toxicants: Lessons learned and future directions. Toxicol In Vitro.

[76] Veranth J.M., Kaser E.G., Veranth M.M., Koch M., Yost G.S. (2007). Cytokine responses of human lung cells (BEAS-2B) treated with micron-sized and nanoparticles of metal oxides compared to soil dusts. Part Fibre Toxicol.

[77] Pickholtz E., Admon D., Izhar U., Berkman N., Levi-Schaffer F. (2011). Dexamethasone and salbutamol stimulate human lung fibroblast proliferation. World Allergy Organ J.

[78] Tanaka S., Yamagishi R., Tsutsui M., Kishida T., Murakami M., Kuroda J., Yoshida T. (2005). Tissue- and dose-dependent alteration of stress-inducible proteins by beta2-adrenoceptor agonist, salbutamol, in rats. J Toxicol Sci.

[79] Rose-John S. (2012). IL-6 trans-signaling via the soluble IL-6 receptor: importance for the pro-inflammatory activities of IL-6. Int J Biol Sci.

[80] Mokra D., Kosutova P., Balentova S., Adamkov M., Mikolka P., Mokry J., Antosova M., Calkovska A. (2016). Effects of budesonide on the lung functions, inflammation and apoptosis in a saline-lavage model of acute lung injury. J Physiol Pharmacol.

[81] Poachanukoon O., Koontongkaew S., Monthanapisut P., Pattanacharoenchai N. (2017). Mometasone Furoate Suppresses PMA-Induced MUC-5AC and MUC-2 Production in Human Airway Epithelial Cells. Tuberc Respir Dis (Seoul).

[82] Takami S., Mizuno T., Oyanagi T., Tadaki H., Suzuki T., Muramatsu K., Takizawa T., Arakawa H. (2012). Glucocorticoids inhibit MUC5AC production induced by transforming growth factor-α in human respiratory cells. Allergol Int.

[83] Komiya K., Kawano S., Suzaki I., Akaba T., Kadota J.-I., Rubin B.K. (2018). Tiotropium inhibits mucin production stimulated by neutrophil elastase but not by IL-13. Pulm Pharmacol Ther.

[84] Arai N., Kondo M., Izumo T., Tamaoki J., Nagai A. (2010). Inhibition of neutrophil elastase-induced goblet cell metaplasia by tiotropium in mice. Eur Respir J.

[85] Zhou Y, Zhang Y, Guo Y, Zhang Y, Xu M, He B. β2-Adrenoceptor involved in smoking-induced airway mucus hypersecretion through β-arrestin-dependent signaling. PLoS One 9(6) (2014) e97788. 10.1371/journal.pone.0097788. PMC4048185 24905583

[86] Kamachi A., Munakata M., Nasuhara Y., Nishimura M., Ohtsuka Y., Amishima M., Takahashi T., Homma Y., Kawakami Y. (2001). Enhancement of goblet cell hyperplasia and airway hyperresponsiveness by salbutamol in a rat model of atopic asthma. Thorax.

[87] Nguyen L.P., Al-Sawalha N.A., Parra S., Pokkunuri I., Omoluabi O., Okulate A.A., Windham Li E., Hazen M., Gonzalez-Granado J.M., Daly C.J., McGrath J.C., Tuvim M.J., Knoll B.J., Dickey B.F., Bond R.A. (2017). beta2-Adrenoceptor signaling in airway epithelial cells promotes eosinophilic inflammation, mucous metaplasia, and airway contractility. Proc Natl Acad Sci U S A.

